# Larvae of five horticulturally important species of *Chrysopodes* (Neuroptera, Chrysopidae): shared generic features, descriptions and keys

**DOI:** 10.3897/zookeys.262.4119

**Published:** 2013-02-04

**Authors:** Patrícia S. Silva, Catherine A. Tauber, Gilberto S. Albuquerque, Maurice J. Tauber

**Affiliations:** 1 Laboratório de Entomologia e Fitopatologia, CCTA, Universidade Estadual do Norte Fluminense, Campos dos Goytacazes, Rio de Janeiro, Brazil 28013-602; 2 Department of Entomology, Comstock Hall, Cornell University, Ithaca, NY 14853-2601 and Department of Entomology, University of California, Davis, CA

**Keywords:** Systematics, immature stages, Neotropical lacewings, comparative morphology

## Abstract

An expanded list of generic level larval characteristics is presented for *Chrysopodes*; it includes a reinterpretation of the mesothoracic and metathoracic structure and setation. Keys, descriptions and images of Semaphoront A (first instar) and Semaphoront B (second and third instars) are offered for identifying five species of *Chrysopodes (Chrysopodes)* that are commonly reported from horticultural habitats in the Neotropical region.

## Abstract (es)

Uma lista expandida das características larvais em nível de gênero é apresentada para *Chrysopodes*, incluindo a reinterpretação da estrutura e das cerdas do mesotórax e metatórax. Chaves, descrições e imagens do semaforonte A (primeiro instar) e semaforonte B (segundo e terceiro instares) são fornecidas para a identificação de cinco espécies de *Chrysopodes (Chrysopodes)* comumente encontradas em habitats hortícolas na região Neotropical.

## Introduction

*Chrysopodes* is one of the main groups of predaceous insects that have value in the biological control of arthropod pests in Neotropical agriculture (Albuquerque et al. 2001, Freitas and Penny 2001, Silva et al. 2007, González Olazo and Heredia 2010). The genus is widespread and relatively large; it occurs throughout all of tropical and subtropical America and presently it consists of 47 species (Tauber et al. 2012). Many species in the genus are commonly found in disturbed habitats, most often in orchards and plantations. One species is reported from the United States (see Tauber 2003, Tauber and Flint 2010), substantially more from Mexico and Central America, and many more from South America.

Navás (1913) described *Chrysopodes* on the basis of the external adult features of a single species. Subsequent researchers included ~30 additional species in the genus, recognized generically distinctive genitalic characteristics, and divided the group into two subgenera: *Chrysopodes* with sickle-shaped mandibles and *Neosuarius* with broadly-tipped mandibles (Adams and Penny 1985, Brooks and Barnard 1990; also see Banks 1945). Thereafter, other species of *Chrysopodes* (*Chrysopodes*) were described (Penny 1998, 2001, 2002, Freitas and Penny 2001, Tauber et al. 2012), and the subgenus *Chrysopodes* (*Neosuarius)* was revised (Tauber 2010). The subgenus *Chrysopodes* (*Chrysopodes*) is currently under revision (Tauber, C. A. in preparation).

Although most of the taxonomic work on the genus *Chrysopodes* has focused on the adult stage, an extensive suite of morphological traits was shown to distinguish *Chrysopodes* larvae from those in other genera of Chrysopini (Tauber 2003). To date, species-specific larval characteristics have been described for two *Chrysopodes* (*Neosuarius*) species: *Chrysopodes (Neosuarius) collaris* (Schneider) and *Chrysopodes (Neosuarius) porterinus* (Navás) (Tauber 2003, Monserrat and Freitas 2005). It is reasonable to expect that further comparative study of the larvae will provide important information for the systematics of *Chrysopodes* and increase the value of this group of natural enemies for ecological investigations and agricultural use.

With the above in mind, we describe and provide images of the larvae of five additional species of *Chrysopodes*. All five species are in the subgenus *Chrysopodes (Chrysopodes)*: *Chrysopodes (Chrysopodes) divisus* (Walker), *Chrysopodes (Chrysopodes) fumosus* Tauber & Albuquerque, *Chrysopodes (Chrysopodes) geayi* (Navás), *Chrysopodes (Chrysopodes) lineafrons* Adams & Penny, and *Chrysopodes (Chrysopodes) spinellus* Adams & Penny. Also, we present keys for identifying the larvae (all instars) of the five species. Prior to doing so, we make some minor corrections and important additions to an earlier list (Tauber 2003) of larval features that distinguish *Chrysopodes*. The genus-level features (i.e., those that are shared by larvae of all *Chrysopodes* species studied to date) are listed on the Appendix.

## Methods

The specimens used in our study were reared from field-collected females. The rearing, preservation, descriptive procedures, and terminology are identical to those published previously (Tauber 2003, http://esa.publisher.ingentaconnect.com/content/esa/aesa/2003/00000096/00000004/art00008 ). We suggest that readers refer to the illustrations and explanatory material in that paper when using the keys, descriptions and images here. Voucher specimens (adult females with their laboratory-reared offspring, and the larval specimens used in the study) are deposited in the Essig Museum, University of California, Berkeley, the insect collection at the Universidade Estadual do Norte Fluminense, Campos dos Goytacazes, and the research collections of the authors.

The earlier study of *Chrysopodes* larval traits (Tauber 2003) was based on laboratory-reared specimens from eight species. Subsequently, two of these “species” were found to be the same; this species is included here [*Chrysopodes (Chrysopodes) spinellus*: Tauber Lots 2001:007, 2002:026]. In addition, three other species from the earlier study are included here [*Chrysopodes (Chrysopodes) divisus*: Tauber Lots 96:017, 96:018, 96:019, 99:020, 99:043, *Chrysopodes (Chrysopodes) geayi*: Tauber Lot 2001:003, previously referred to as “*pulchella*”, and *Chrysopodes (Chrysopodes) fumosus*: Tauber Lot 2002:021]. Two of the remaining lots from the earlier study will be described elsewhere [Tauber Lots 96:006, 99:037], and one [*Chrysopodes (Neosuarius) collaris*] was described earlier (Tauber 2003).

In our previous work, we have used two terms “submedian setae” (e.g., Mantoanelli et al. 2011) and “submesal setae” (e.g., Tauber et al. 2011) to refer to the dorsal abdominal setae that Tsukaguchi (1995) termed “submedian setae”. Here, to be consistent with Tsukaguchi, we use one term, “submedian setae”.

It is noteworthy that bilateral asymmetry in setal numbers is common, and that specimens occasionally exhibit variation in the numbers and sizes of setae (especially in the dorsal thoracic setae and the submedian abdominal setae) of all instars. The numbers presented here reflect this asymmetry. Also, in our descriptions, unless stated otherwise, all setae, other than the submedian setae, are smooth and pointed (not thorny, hooked or blunt).

## Shared Generic Characteristics

The chrysopid life cycle includes a larval stage with three instars. The first instar differs markedly in structure, setation and often coloration from the other two instars, which differ from each other only in minor ways, largely related to size. Thus, for taxonomic purposes, the first instar constitutes Semaphoront A, whereas Semaphoront B includes both the second and third instars and Semaphoront C encompasses all instars (= the larva) (See Wheeler 1990). We use the term “semaphoront” in our descriptions because of its systematic and phylogenetic value. Specifically: (i) the term highlights the relative degree of morphological change that occurs with each instar during metamorphosis, (ii) it reflects accurately the relative value of the three chrysopid instars to phylogenetic analysis, and (iii) the commonality of the pattern of variation among semaphoronts across chrysopid taxa, and indeed taxa in other insect orders (see Wheeler 1990), itself is of considerable biological interest.

Chrysopid genera fall into two general categories: those with “naked” larvae and those with “trash-carrying” larvae. *Chrysopodes* larvae are typical examples of the latter – that is, they have compact, globose bodies, hooked abdominal setae, and well developed thoracic and abdominal tubercles that bear elongate setae adapted for carrying small pieces of plant or animal debris. Furthermore, *Chrysopodes* larvae express a unique set of morphological and setal characters that distinguishes them from the larvae of other trash-carrying genera (Tauber 2003; for additional comparisons, see Díaz-Aranda and Monserrat 1995, Tsukaguchi 1995, Monserrat and Díaz-Aranda 2012).

**Figure 1.**
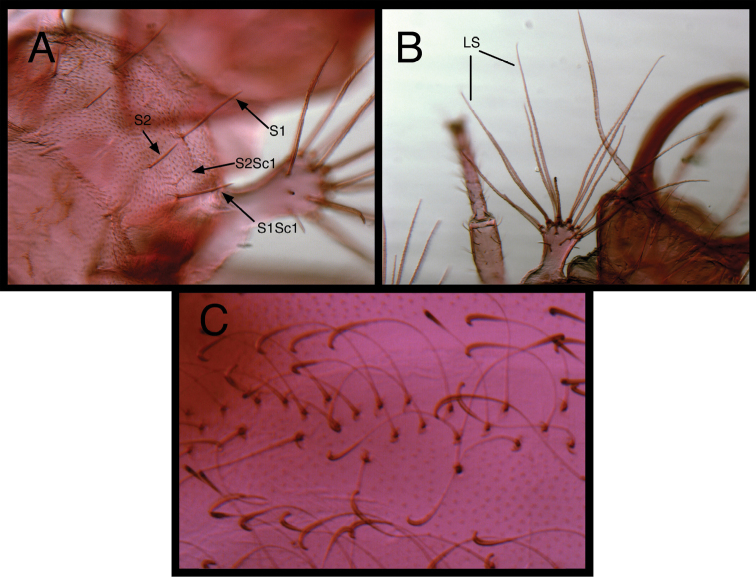
Examples of dorsal setae that typify *Chrysopodes* larvae (laboratory-reared third instar *Chrysopodes divisus*) **A** Two types of dorsal prothoracic setae: (1) thorny, pointed or blunt (**S1, S1Sc1**) and smooth, pointed (**S2, S2Sc1**) [Note the position of the setae relative to the dark sclerite.] **B** Elongate, robust, thorny, blunt or spatulate setae on lateral tubercle (**LS**) **C** Elongate, smooth, hooked submedian setae of anterior abdominal segments [Note the narrowed and bent middle section and the enlarged, laterally flattened, hooked terminus.].

In general, the species studied here exhibit all of the larval characteristics proposed earlier to typify *Chrysopodes* (Tauber 2003); moreover, several additional characteristic features were discovered during the current study. Thus, we provide an up-dated list of shared *Chrysopodes* generic-level characteristics (Appendix); those that are new as a result of the current study are marked with an asterisk. Among the most distinctive features that were previously unreported are the uniquely shaped submedian setae (SMS) on the anterior abdominal segments of all instars (Fig. 1C). These long, smooth, hooked setae are slender and bent throughout the midregion, but their hooked tips are robust, rigid and laterally compressed. We have not seen this type of seta on larvae of other neotropical Chrysopini.

## Keys to larvae of five *Chrysopodes (Chrysopodes)* spp. commonly found in Brazilian fruit orchards

Note: To identify cephalic setae, see Fig. 5 on page 477 of Tauber (2003). For body setae, see Fig. 10 on page 482 (Semaphoront A) and Figs 6 and 8 on pages 478 and 480 (Semaphoront B) of the same article – http://esa.publisher.ingentaconnect.com/content/esa/aesa/2003/00000096/00000004/art00008

### First instar (Semaphoront A)

**Table:** 

1	Head predominantly brown; epicranial marking entire (with mesal and lateral arms wholly or partly confluent) and fused mesally (Figs 2A, 2D); abdominal segments A1-A5 each with total of more than 14 long, hooked, dorsal setae (spiracular and submedian setae)	2
–	Head predominantly white to cream-colored; epicranial marking consisting of two longitudinally elongate brown stripes, with mesal and lateral arms completely separate (Figs 2B-C, 2E); abdominal segments A1-A5 each with total of 14 long, hooked, dorsal setae (spiracular and submedian setae)	3
2	Cranium with two dorsal setae (S1 and S11) thorny (Fig. 5A in Tauber 2003); dorsum of abdominal segments A1-A5 each with total of more than 30 elongate, smooth, hooked setae (submedian and spiracular setae) (Fig. 5C)	*Chrysopodes (Chrysopodes) divisus*
–	Cranium with four dorsal setae (S1, S4, S6 and S11) thorny; dorsum of abdominal segments A1-A5 each with total of 16–20 elongate, smooth, hooked setae (submedian and spiracular setae) (Fig. 19C)	*Chrysopodes (Chrysopodes) lineafrons*
3	Paired frontal marking with posterior ends straight, not meeting mesally (Fig. 2B)	*Chrysopodes (Chrysopodes) fumosus*
–	Paired frontal marking with posterior ends curved inward, meeting mesally (Fig. 2C, 2E)	4
4	More than three (usually six) cranial setae (S1, S3, S4, S5, S6, S11) thorny (Fig. 5A in Tauber 2003); large lateral tubercles on thorax and abdomen with setae (LS) light amber to light brown (Fig. 23C)	*Chrysopodes (Chrysopodes) spinellus*
–	Only two or three cranial setae (S1, S11, sometimes S4) thorny; large lateral tubercles on thorax and abdomen with setae (LS) dark brown (Fig. 14C)	*Chrysopodes (Chrysopodes) geayi*

### Second and third instars (Semaphoront B)

**Table:** 

1	Epicranial mark broad, with two arms wholly or partly confluent; paired frontal markings broadly fused mesally (Figs 3A, 3D, 4A, 4D)	2
–	Epicranial mark consisting of two paired longitudinal stripes (= mesal and lateral arms); paired frontal markings close to each other, but separate, except sometimes posterior tips curve and meet mesally (Figs 3B-C, 3E, 4B-C, 4E)	3
2	Mesal and lateral arms of epicranial marking fully confluent throughout (Figs 3A, 4A); cranial seta S1 smooth; pronotum with sparse covering of largely transparent spinules; metathorax with posterior fold bearing transverse row of 14–15 long, thorny setae arising from robust, brown chalazae and one pair of smooth, lateral setae arising from smaller chalazae (Figs 6C, 7A, 8A)	*Chrysopodes (Chrysopodes) divisus*
–	Mesal and lateral arms of epicranial marking confluent basally, separate distally (Figs 3D, 4D); cranial seta S1 thorny; pronotum with dense covering of dark brown spinules; metathorax with posterior fold bearing transverse row of 12 to 13 long, thorny setae arising from robust, brown chalazae (Figs 20C, 21A, 22A)	*Chrysopodes (Chrysopodes) lineafrons*
3	Paired frontal marking with posterior ends straight, not meeting mesally (Figs 3B, 4B); all cranial setae smooth	*Chrysopodes (Chrysopodes) fumosus*
–	Paired frontal marking with posterior ends curving and meeting mesally (Figs 3C, 3E, 4C, 4E); cranial seta S1 thorny	4
4	Cranium with three to four pairs of small secondary setae between S1 and S4 (Fig. 5B in Tauber 2003); pronotum with paired sclerite (Sc1) light brown to transparent, forked basally with mesal arm very faint, lateral arm light brown to transparent; midline of pronotum with only one sclerite (Sc2), no secondary sclerites (Figs 3E, 4E)	*Chrysopodes (Chrysopodes) spinellus*
–	Cranium without secondary setae (Fig. 5A in Tauber 2003); pronotum with paired sclerite (Sc1) dark brown, forked basally into distinct mesal and lateral arms that extend around base of lateral tubercles; midline of pronotum with several secondary sclerites in addition to Sc2 (L2: one to two; L3: five to seven) (Figs 3C, 4C)	*Chrysopodes (Chrysopodes) geayi*

## Larval descriptions

Because the Appendix lists the generic-level characteristics that are shared by all *Chrysopodes* larvae studied to date, the individual descriptions presented here are restricted to those larval characteristics that distinguish the species.

### Chrysopodes (Chrysopodes) divisus

(Walker, 1853)

http://species-id.net/wiki/Chrysopodes_divisus

**Figure 2.**
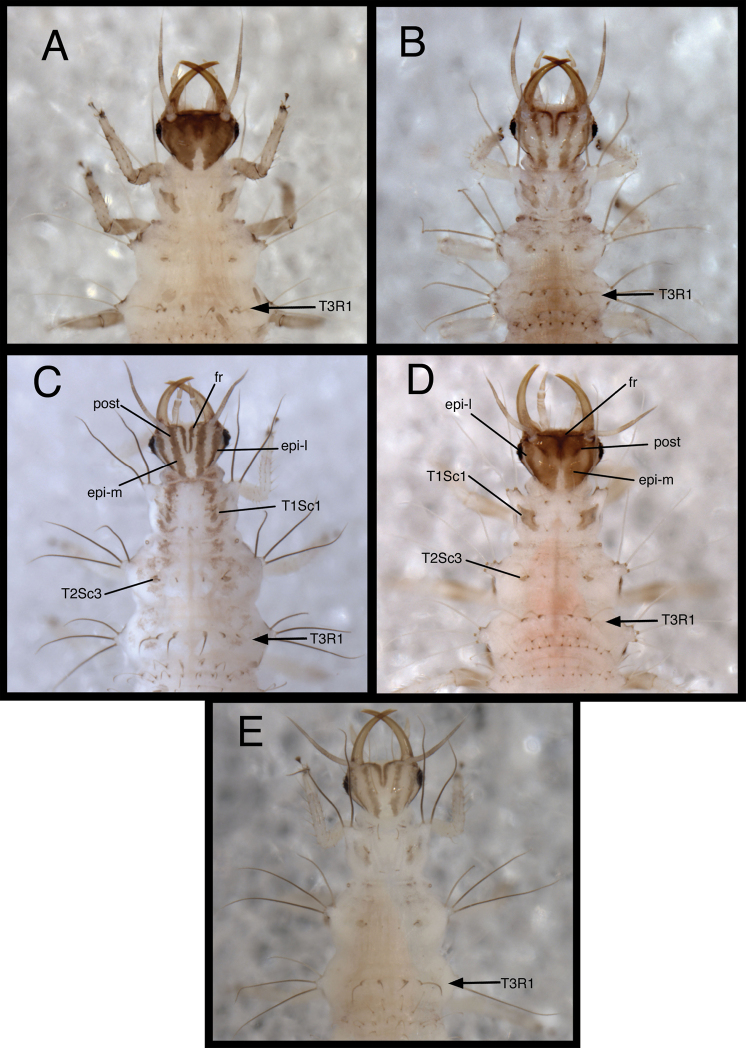
Head and thorax, dorsal, first instar **A** *Chrysopodes (Chrysopodes) divisus* **B** *Chrysopodes (Chrysopodes) fumosus* **C** *Chrysopodes (Chrysopodes) geayi* **D** *Chrysopodes (Chrysopodes) lineafrons* **E** *Chrysopodes (Chrysopodes) spinellus*. *Abbreviations*: **epi-l** epicranial marking, lateral arm **epi-m** epicranial marking, mesal arm **fr** frontal marking **post** postfrontal marking **T1Sc1** first primary prothoracic sclerite **T2Sc3** third primary mesothoracic sclerite **T3R1** metathoracic row of robust, thorny setae.

Figs 2 [Fig F3] [Fig F4] [Fig F5] [Fig F6] [Fig F7] [Fig F8] –9

#### Discussion.

*Chrysopodes divisus* is probably one of the most common and widespread of the *Chrysopodes* species. It has a large number of synonyms (see Adams and Penny 1985), and originally it was placed in the subgenus *Chrysopodes (Neosuarius)*. Recently it was moved to *Chrysopodes (Chrysopodes)* on the basis of adult characteristics (Tauber 2010).

Adults of *Chrysopodes (Chrysopodes) divisus* are recognized by their relatively narrow costal cells, dark gradate veins, facial markings and very distinctive male and female genitalia. They can be identified using current keys and redescriptions (Adams and Penny 1985, Freitas and Penny 2001).

#### Known geographic distribution.

Argentina, Brazil, British Guiana, Colombia, Cuba, Jamaica, Paraguay, Peru, Uruguay, and Venezuela (Adams and Penny 1985, Freitas and Penny 2001).

#### Larval diagnosis.

*Chrysopodes (Chrysopodes) divisus* larvae (all instars) are relatively short, compact, and rotund. Their bodies are white to light cream-colored, with brown to light brown prothoracic sclerites; most of the long setae are cream-colored to light brown. The extensive brown head markings of *Chrysopodes (Chrysopodes) divisus* (all instars) are similar to those of *Chrysopodes (Chrysopodes) lineafrons* in that: (i) The confluent frontal markings, together with the dark brown intermandibular region, form an extensive dark brown, triangular to T-shaped mark on the anterior region of the head. (ii) The mesal and lateral arms of the epicranial markings are completely or partially confluent; together with the dark brown postfrontal markings, they cover most of the posterior and mesolateral regions of the head.

The first instar of *Chrysopodes (Chrysopodes) divisus* is distinguished from *Chrysopodes (Chrysopodes) lineafrons* by the lack of thorns on all cranial setae other than S1 and S11, metathorax with a large number of setae (n = 6–7) in row (R1) on posterior fold, and a large number of abdominal SMS on segments A1 to A5 (n > 30). Second and third instars (Semaphoront B) are characterizedby cranial setae that are all without thorns, prothorax that is white to cream-colored and without a conspicuous covering of brown spinules, and metathorax that has a large number of setae (n = 14–15) in row (R1), which, in the L3, is flanked laterally by a pair of long, smooth setae.

#### First instar.

(Semaphoront A). *Body* (Fig. 5A) short, globose, compact in shape, 2.5–2.9 mm long. *Head* (Figs 2A, 5B) 0.38–0.39 mm wide; mandibles 0.35–0.39 mm long (ratio, mandible length : head width = 0.95–1.02 : 1). Dorsum of cranium predominantly brown, with wedge-shaped, white area posteromesally. Epicranial marking entire, light brown mesally, darker brown laterally. Postfrontal marking darker brown than most of epicranial marking, contiguous basally with distal margin of epicranial marking, extending to anterior base of antenna. Frontal marking paired, but fused mesally, dark brown anteriorly, narrow, extending anteriorly from midregion of cranium, bending toward inner basal margin of mandible. Intermandibular, clypeal area brown. Cranial setae light amber; S1, S11 long, thorny, others smooth, shorter.

Gena, ventral margin of cranium brown; genal marking with small white spot behind eye. Labial palpus tinged with light brown, slightly darker distally. Mandibles amber, with brown basolateral spot. Antenna with scape brownish, pedicel white basally, brown distally, flagellum light brown.

*Thorax* (Figs 2A, 5A) mostly cream to white, with sclerotized structures light to very light brown; episternum light brown. Legs white, with base of coxa light brown, femur (especially distal half) tinged with brown, dorsum of tibia, tarsus tinged with light brown; tarsal claws, empodia brown. LS cream to white; other setae white to cream.

T1: Row of three very small setae (R1) at anteromesal base of LTs. Sc1 brown mesally, basolaterally, with cream colored interior; S2Sc1 small, immediately above S1Sc1. S1 long, S3 intermediate-length. T2: Spiracle with lips of atrium protruding above integumental surface. Sc1, Sc2, transparent; Sc3 marked with light brown; S2Sc3 variable, medium-length to long; S2 smaller. T3: S1Sc1, S2Sc1 present, S1Sc2 very small, sometimes absent, S2Sc2 usually present. Posterior fold with row (R1) of six (sometimes seven) long, thorny, pointed setae on chalazae with ovate, light brown marks anteriorly; lateral two chalazae on each side juxtaposed.

*Abdomen* (Figs 5C-D) white to cream-colored, with LTs, LDTs tinged with light brown.

A1: Dorsum with ten to twelve SMS in single anterior row, with 34–42 SMS in double to triple row posteriorly; spiracles at end of posterior row, without distinguishable SSp. A2: Dorsum with ~10 SMS in single anterior row, with ~44 SMS in double to triple posterior row; anterior row bending posteriorly at each end, coalescing with setae in posterior row; spiracle at end of posterior row, with SSp near anterodistal margin. A3-A5: Dorsum with ~8 (A3, A4) or 6–7 (A5) SMS in single anterior row, with ~36 (A3), ~32 (A4), ~26 (A5) SMS in double to triple posterior row; anterior row bending posteriorly at each end, coalescing with posterior row; spiracle with SSp anteroventrally. A6: Anterior region with four SMS; spiracle with SSp mesally. A7: Anterior region with pair of microsetae; spiracle with SSp mesally. A8: Venter with two pairs of medium-length setae posteriorly, one pair of short setae slightly anteriorly.

#### Second and third instars.

(Semaphoront B). *Body* (Figs 6E, 7A–B) length 3.3–3.4 mm (L2), 5.7–6.3 mm (L3); surface white to cream-colored, with light to dark brown integumental spinules somewhat dense, dark on pronotum; primary pronotal, mesonotal sclerites brown to dark brown, other dorsal marks small, brown; sclerites around coxae dark brown, abdomen with light to very light brown stripe laterally.

**Figure 3. F3:**
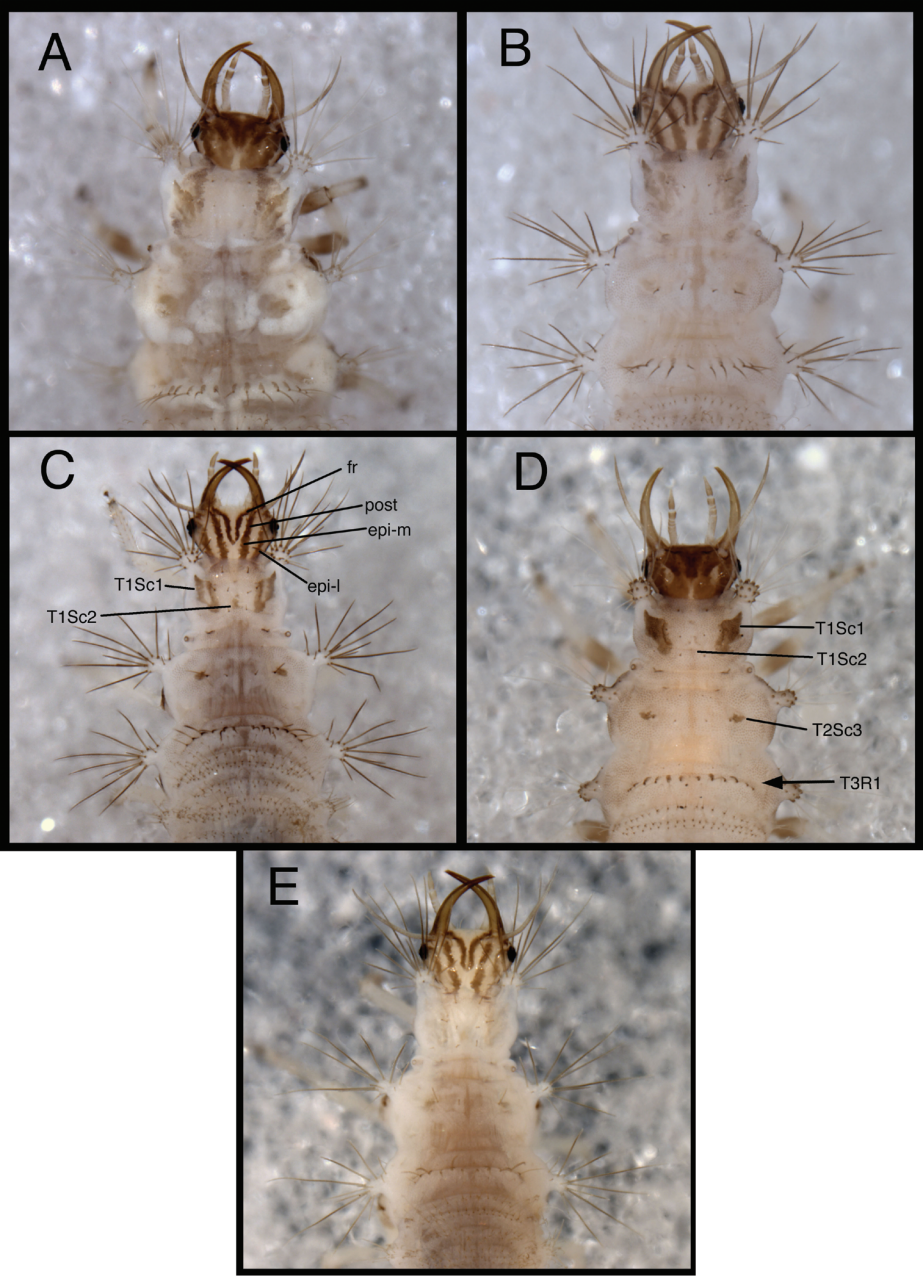
Head and thorax, dorsal, second instar **A** *Chrysopodes (Chrysopodes) divisus* **B** *Chrysopodes (Chrysopodes) fumosus* **C** *Chrysopodes (Chrysopodes) geayi* **D** *Chrysopodes (Chrysopodes) lineafrons* **E** *Chrysopodes (Chrysopodes) spinellus*. *Abbreviations*: **epi-l** epicranial marking, lateral arm **epi-m** epicranial marking, mesal arm **fr** frontal marking **post** postfrontal marking **T1Sc1, T1Sc2** first and second primary prothoracic sclerites **T2Sc3** third primary mesothoracic sclerite **T3R1** metathoracic row of robust, thorny setae.

**Figure 4. F4:**
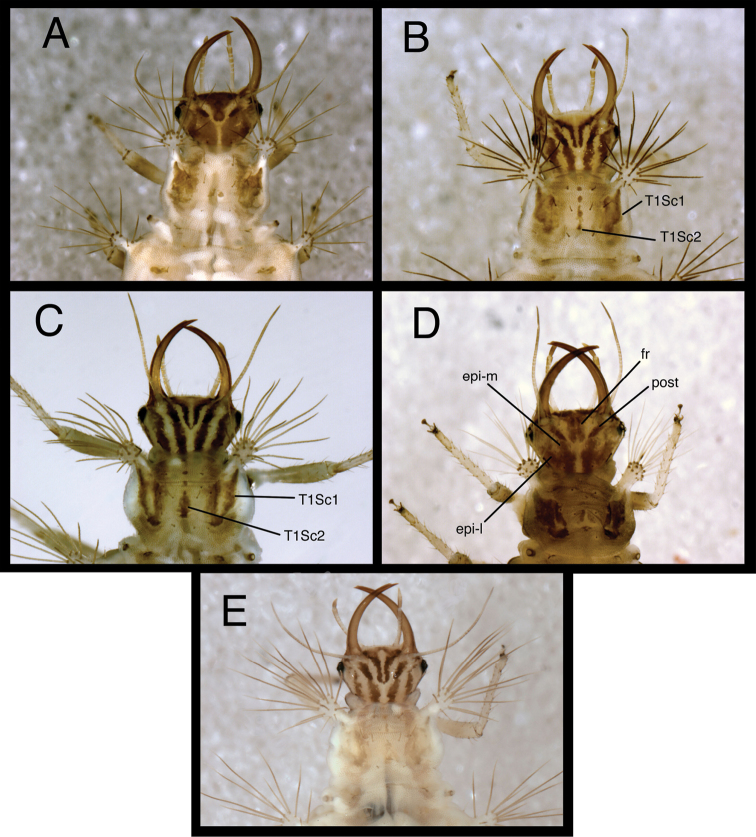
Head and thorax, dorsal, third instar **A** *Chrysopodes (Chrysopodes) divisus* **B** *Chrysopodes (Chrysopodes) fumosus* **C** *Chrysopodes (Chrysopodes) geayi* **D** *Chrysopodes (Chrysopodes) lineafrons* **E** *Chrysopodes (Chrysopodes) spinellus*. *Abbreviations*: **epi-l** epicranial marking, lateral arm **epi-m** epicranial marking, mesal arm **fr** frontal marking **post** postfrontal marking **T1Sc1, T1Sc2** first and second primary prothoracic sclerites.

*Head* (Figs 3A, 4A, 6A–B, 7C–D) cream-colored, with extensive brown and dark brown markings. Epicranial marking undivided, with mesal and lateral arms distinguishable, but broadly connected throughout, both arms in contact with posterior margin of head; lateral arm dark brown basally, lighter distally, extending from distal ~one-fourth of posterior cranial suture to lateral base of mandible; mesal arm light brown, extending from base of head, becoming confluent with postfrontal marking. Postfrontal marking very dark brown, narrow throughout, extending to inner base of scape. Frontal marking dark brown, with left, right arms contiguous with each other and with intermandibular marking, forming broad, triangular, dark brown anterior marking. Clypeolabral region distal to anterior marking cream-colored. Gena cream-colored, with brown marking basally, becoming lighter brown, forking near midregion, extending to base of eye. Mandible, maxilla dark brown laterally and distally, amber mesally, base with dark brown mesal mark. Labial palpus: basal segment cream-colored with light brown mesally; mesal segment ringed with light brown laterally, cream-colored mesally, with terminal subsegment brown; terminal segment dark brown basally, light brown distally. Antenna: scape, basal, mesal sections of pedicel cream-colored to amber, distal section of pedicel very light brown, flagellum light brown. Venter cream-colored to white; margin of cranium brown to dark brown; cardo, stipes brown; base of mentum with light brown patch.

**Figure 5. F5:**
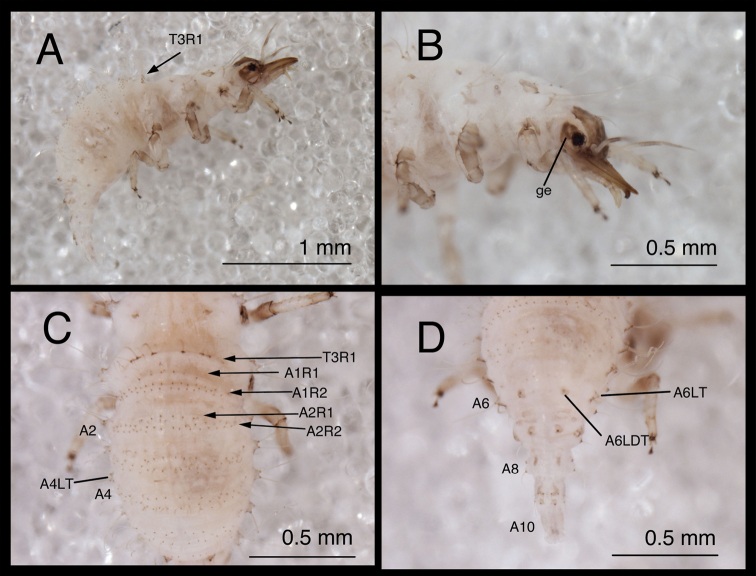
*Chrysopodes (Chrysopodes) divisus*, first instar **A** Habitus, lateral **B** Head, lateral **C** Abdominal segments A1 to A5, dorsal **D** Abdominal segments A6 to A10, dorsal. *Abbreviations*: **A2, A4, A6, A8, A10** abdominal segments **A1R1, A1R2** anterior and posterior rows of submedian setae (SMS) on first abdominal segment **A2R1, A2R2** anterior and posterior rows of SMS on second abdominal segment **A4LT** lateral tubercle on fourth abdominal segment **A6LDT, A6LT** laterodorsal tubercle, lateral tubercle on sixth abdominal segment **ge** genal marking **T3R1** row of long, sturdy, thorny setae on raised posterior fold of metathorax.

**Figure 6. F6:**
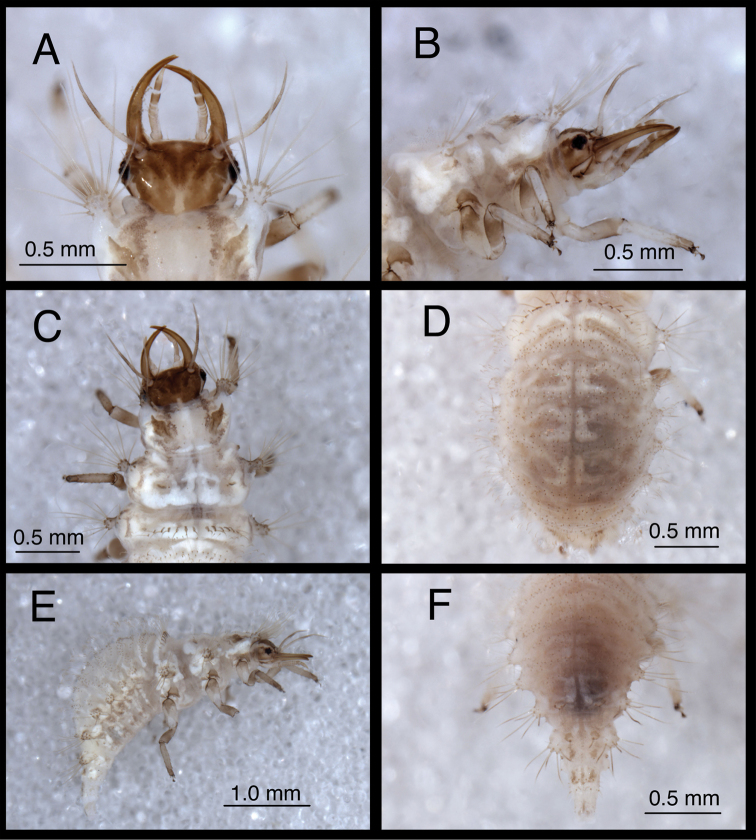
*Chrysopodes (Chrysopodes) divisus*, second instar **A** Head, dorsal **B** Head, lateral **C** Head and thorax, dorsal **D** Abdominal segments A1 to A5, dorsal **E** Habitus, lateral **F** Abdominal segments A6 to A10, dorsal.

All cephalic setae smooth, pointed; S1 slightly robust, medium length, S11 long, S2–10, S12 short to medium length; Vx setae fairly long, robust; with one to two pairs of secondary setae. Anterior margin of head protruding, straight with angled lateral margins; mesal pair of anterior setae much longer than two lateral pairs.

Head width across eyes, 0.53–0.59 mm (L2), 0.78–0.83 mm (L3); mandible length, 0.52–0.56 mm (L2), 0.81–0.90 mm (L3); ratio mandible length to head width = 0.92–1.0 : 1 (L2), 1.0–1.1 : 1 (L3). Tip of mandible with six teeth mesally.

Cervix brownish dorsally, slightly darker laterally; venter cream-colored mesally, light brown laterally; white ventrally.

**Figure 7. F7:**
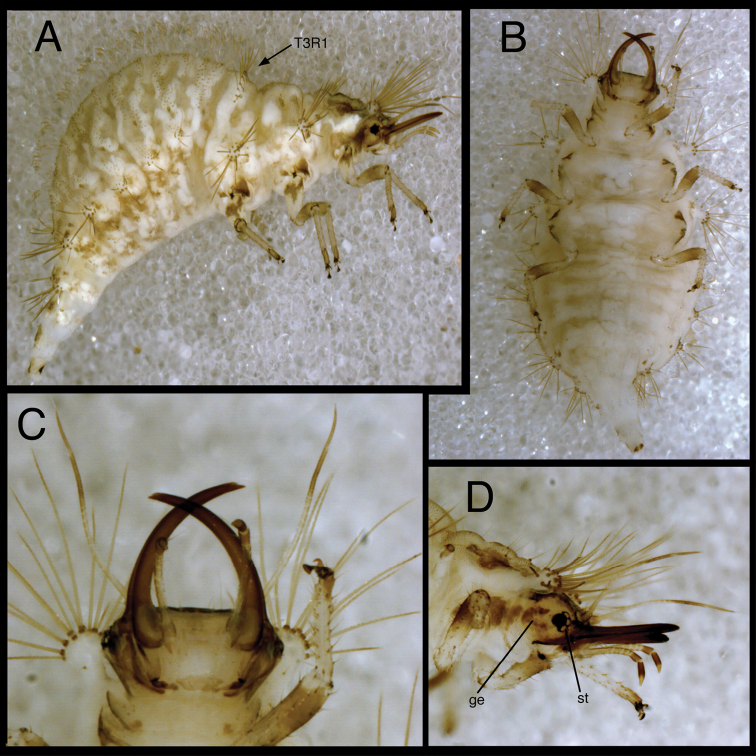
*Chrysopodes (Chrysopodes) divisus*, third instar **A** Habitus, lateral **B** Habitus, ventral **C** Head, ventral **D** Head, lateral. *Abbreviations*: **ge** genal marking **st** stemmata **T3R1** row of long, sturdy, thorny setae on raised posterior fold of metathorax.

**Figure 8. F8:**
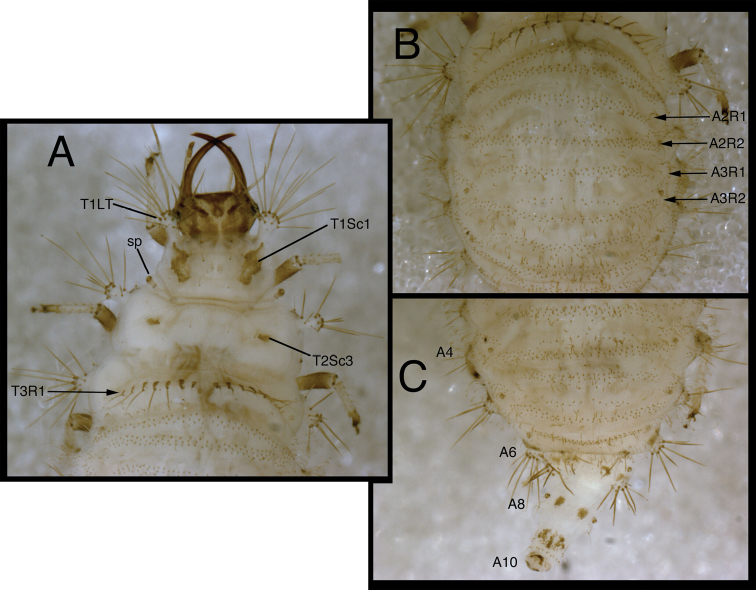
*Chrysopodes (Chrysopodes) divisus*, third instar **A** Thorax, dorsal **B** Abdominal segments A1 to A5, dorsal **C** Abdominal segments A6 to A10, dorsal. *Abbreviations*: **A4, A6, A8, A10** abdominal segments **A2R1** double row of submedian setae (SMS) on anterior fold of second abdominal segment **A2R2** double/triple row of SMS on posterior fold of second abdominal segment **A3R1** double row of SMS on anterior fold of third abdominal segment **A3R2** double/triple row of SMS on posterior fold of third abdominal segment **sp** spiracle (on anterior subsegment of mesothorax) **T1LT** prothoracic lateral tubercle **T1Sc1** first primary prothoracic sclerite **T2Sc3** third primary mesothoracic sclerite **T3R1** row of long, sturdy, thorny setae on raised posterior fold of metathorax.

*Thorax* (Figs 3A, 4A, 6B–C, 6E, 7A–B, 8A) white to cream-colored; dorsum, especially pronotum tinged with brown, the darkness of which depends on density, color of integumental spinules; with sclerites, markings brown; LTs white to cream-colored, with LS amber. Venter white to cream-colored, unmarked. Legs: coxa white, with brown on basodorsal surface; trochanter cream-colored to white, base of femur cream-colored, becoming brownish mesally, cream-colored at tip; tibia white with brown setae; tarsus tinged with light brown; empodium brown; claws amber.

T1: LT with 14–17 (L2), 17–19 (L3) LS. Sc1 large, rhomboid, extending around posterior base of LT, darker brown laterally than mesally. Sc2 triangular, appearing as paired brown marks, with two small, irregularly shaped sclerites (brown) above; sometimes small brown spots anteriorly. S2, S3 smooth. T2: Anterior sclerite (Sc1) light brown to brown; spiracles on prominent protuberances. Posterior subsegment with Sc2 light brown; Sc3 pronounced, brown. LT with 12–13 (L2), 16–19 (L3) LS. T3: Sc1 transparent. LT with 11–13 (L2), 13–18 (L3) LS. Posterior fold with 14 to 15 robust, thorny setae; L3 sometimes with additional pair of long, smooth, pointed setae laterally, arising from smaller chalazae.

*Abdomen* (Figs 6D–F, 7A–B, 8B–C) white to cream-colored, tinged with light brown, with small light brown to brown spots anterior to bases of LTs; spots more diffuse on A5-A8; white fat-body visible beneath integument; setae mostly light brown to amber-colored. A6, A7 each with pair of large, dark brown marks surrounding LDTs. A8 with pair of dark brown marks mesal to spiracles; A9 with pair of large, dark brown marks anterolaterally, dark brown mark mesally; A10 with inverted U-shaped, dark brown mark. Venter white to cream-colored, unmarked, except with some light brown pigmentation ventrolaterally on S5-S9 (some specimens); tip of A10 with pair of small, abutting, triangular dark brown marks.

A1: Dorsum with ~78–88 (L2), 142–172 (L3) SMS in two double-triple transverse bands between spiracles. A2-A5: Dorsum with 64–112 (L2), 120–196 (L3) SMS in two broad transverse bands. LTs each with 11–21 (L2), 29–40 (L3) LS: one to five robust, thorny, blunt to spatulate LS on distal surface; remaining LS long, smooth, hooked, in large patch on dorsal surface. A6: Dorsum with transverse band of 20–30 (L2), 56–66 (L3) SMS across anterior of segment; midsection with one to two pairs of smooth setae, mesal pair hooked (similar to SMS), lateral pair short, pointed. LT with nine to eleven (L2), ten to eleven (L3) LS of various sizes. A7: Dorsum with one to two pairs of very small setae (S1, S2) anteriorly, between spiracles; LDTs each with one medium-length, robust, thorny, blunt to spatulate LDS, one to two smaller, smooth, pointed LDS; pair of very small setae between LDTs. LT with ten to 13 (L2), ten to 14 (L3) LS of various sizes. A8: Anterior region with one to two pairs of very small setae (S1, S2). Venter with pair of medium-length setae between LTs, two to three smaller setae slightly anteroventral to LTs. A9: Dorsum with one pair of very small setae anteriorly. Middle and posterior regions with two transverse rings of setae extending around segment; each ring with ~14–16 short to medium-length setae, several in each ring robust. A10: Dorsum with several pairs of small setae on V-shaped anterior sclerites, one slightly anterior to terminus. Two pairs of robust lateral setae. Venter with ~five pairs of small setae in V-shaped pattern, posterior row of microsetae anterior to terminus.

#### Egg.

At oviposition, light yellowish green to green, with white micropyle; ovoid, 0.82 to 1.06 mm long, 0.36 to 0.43 mm wide. Stalk smooth, hyaline, 2.89 to 6.80 mm long.

#### Larval specimens examined.

Numerous lots, each originating from a single gravid female collected in **Brazil, Bahia**: Cruz das Almas, VI-19–96 (Tauber Lots 96:017C, 96:018B, 96:019B, 96:019D); Camacan, Reserva Serra Bonita, 800 m., X-3 to 7–2005 (Tauber Lot 2005:032). **Distrito Federal,** Brasília, X-22 and 23–2003 (Tauber Lots 2003:035, 2003:036, 2003:038). **Minas Gerais,** Lavras, UFLA Campus, coffee orchard, X-12–2005 (Tauber Lot 2005:020); Lavras, Parque Ecológico Cachoeiras do Rio Bonito, X-14-2005 (Tauber Lot 2005:028). **Rio de Janeiro**: Conceição de Macabu, Santo Agostinho, V-21-2002 (Tauber Lot 2002:020); Conceição de Macabu, Fazenda Carrapeta, II-28-2002, IV-29 to V-6-2003 (Albuquerque Lot 2002:05, Tauber Lot 2003:007); Santa Maria Madalena, Terras Frias, III-30-1999 (Tauber Lot 99:043). **Rio Grande do Sul:** Cachoeira do Sul, São Nicolau, I-16-2007 (Tauber Lots 2007:023A, 2007:023B). Two field-collected L3 from RJ, Conceição de Macabu, Santo Agostinho, V-2-2003.

#### Biology.

Adults and larvae of this species were collected on shrubs in disturbed, dry forest habitats. Adults are agile; they exhibit fast, evasive flight, usually inward toward the interior of the bush or tree.

Based on the following observations, we think that *Chrysopodes (Chrysopodes) divisus* adults may enter a diapause-mediated dormancy. Adults collected during the spring in Minas Gerais (Parque Ecológico Cachoeiras do Rio Bonito) were yellow to yellowish brown, and they had a greasy appearance. In addition, their prothoracic stripes were pronounced, broad and relatively dark reddish brown. In the lab, reproduction by these adults did not occur until after they had been held under warm, long-day conditions with ample food and water for over a week. Moreover, reproduction was correlated with the assumption of bright green coloration, the loss of some of the reddish brown coloration on the thorax, and a narrowing of the prothoracic stripes. In other Chrysopini adults, e.g., species of *Chrysoperla*, changing behavioral and color patterns like those described for *Chrysopodes (Chrysopodes) divisus* have been shown to be part of the diapause syndrome (Tauber et al. 1986).

**Figure 9.**
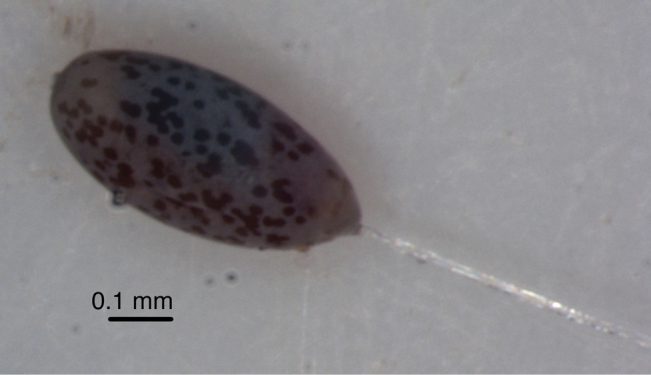
*Chrysopodes (Chrysopodes) divisus*, egg (~5 days old).

In the lab, eggs from all the lots listed above were deposited separately (with isolated stalks), in small groups with no particular pattern; the stalks were sticky, but without droplets. During the first 24 hours after oviposition, the eggs were bright yellowish green to green, without spots. On the second day, they began to develop a bluish brown color, with grey or brownish mottling which became more pronounced as hatching approached (Fig. 9). At 24 ± 1°C, hatching occurred within six to eight days (lots from three females collected in Cruz das Almas, n = 17 – 48 eggs/female).

In one case, a small proportion (n = 2 of 28) of the eggs laid by a female from Minas Gerais had a prolonged incubation period (approximately one week to ten days longer than the usual six- to eight-day incubation period). They were a dark bluish brown color during the period of delay. The cause of the prolongation is unknown; however, the resulting larvae developed normally and appeared healthy.

Larvae of *Chrysopodes (Chrysopodes) divisus* carry pieces of woody plant material and other dry debris on their backs. In the rearings (24±1°C) from three females collected at Cruz das Almas, development of the various stages required: L1, 5–8 days; L2, 5–7 days; L3, 5–9 days; cocoon, 16–20 days; complete development from oviposition to adult emergence, 40–48 days. Among the offspring of each of the three females, the sex ratio was approximately 1 : 1 (n = 19–33 individuals / female). The developmental and reproductive responses of *Chrysopodes (Chrysopodes) divisus* to a broad range of temperatures are reported elsewhere (Silva et al. in prep.).

### Chrysopodes (Chrysopodes) fumosus

Tauber & Albuquerque, 2012

http://species-id.net/wiki/Chrysopodes_fumosus

Figs 2 [Fig F3] –4 , 10 [Fig F12] –13

#### Discussion.

Adults of this recently described species are readily distinguished from other *Chrysopodes* species. Specifically, the *Chrysopodes (Chrysopodes) fumosus* forewings have venation that is extensively crassate and uniquely patterned; the membrane surrounding many of the crossveins is heavily fumose – thus, the species name. Both the male and female genitalia are distinctive and should be examined for accurate identification of the species (see description and figures in Tauber et al. 2012).

#### Known geographic distribution.

Brazil, Venezuela (Tauber et al. 2012).

#### Larval diagnosis.

Like the larvae of *Chrysopodes (Chrysopodes) geayi* and *Chrysopodes (Chrysopodes) spinellus*, *Chrysopodes (Chrysopodes) fumosus* larvae have largely white to cream-colored heads with brown, longitudinally elongate and divided epicranial markings; the intermandibular and clypeal regions are unmarked. Their frontal markings are distinguished from those of the other two species in that their posterior ends are straight and do not curve or connect mesally. *Chrysopodes (Chrysopodes) fumosus* Semaphoront A also differs from those of the other two species in that it has only two thorny cranial setae (S1, S11). And, *Chrysopodes (Chrysopodes) fumosus* Semaphoront B is distinguished from *Chrysopodes (Chrysopodes) geayi* and *Chrysopodes (Chrysopodes) spinellus* in that all of its cranial setae (including S1) are without thorns.

#### First instar.

**Figure 10.**
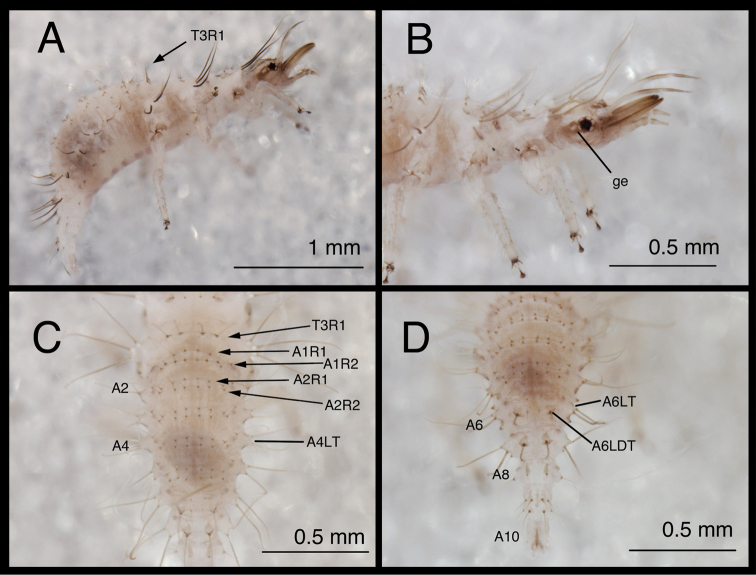
*Chrysopodes (Chrysopodes) fumosus*, first instar **A** Habitus, lateral **B** Head, lateral **C** Abdominal segments A1 to A5, dorsal **D** Abdominal segments A6 to A10, dorsal. *Abbreviations*: **A2, A4, A6, A8, A10** abdominal segments **A1R1, A1R2** anterior and posterior rows of submedian setae (SMS) on first abdominal segment **A2R1, A2R2** anterior and posterior rows of SMS on second abdominal segment **A4LT** lateral tubercle on fourth abdominal segment **A6LDT, A6LT** laterodorsal tubercle, lateral tubercle on sixth abdominal segment **ge** genal marking **T3R1** row of long, sturdy, thorny setae on raised posterior fold of metathorax.

(Semaphoront A). *Body* (Fig. 10A) 2.3–2.8 mm long. Surface predominantly white to cream-colored, with some, small, light brown marks, light dusting of brown, especially on sides and venter.

*Head* (Figs 2B, 10B) 0.39–0.41 mm wide; mandibles 0.36–0.38 mm long (ratio, mandible length : head width = 0.86–0.96 : 1). Cranium mostly white to cream colored, with elongate, narrow, light brown markings. Epicranial marking consisting of two, unconnected, narrow, longitudinally elongated stripes (arms); mesal arm contiguous with postfrontal marking, extending from base of cranium to anteromesal margin of antennal socket; lateral arm lighter brown, more diffuse than mesal arm, extending from posterolateral margin of cranial suture approximately to distal base of mandible. Postfrontal marking indistinguishable, fused with mesal arm of epicranial marking. Frontal marking narrow, extending from midregion of cranium anteriorly to level of antennal socket, then curving laterally toward lighter brown mark at mesal margin of mandibular base; posteromesal ends of marking curving inward, fusing narrowly at tip. Intermandibular, clypeal areas white. Cranial setae amber to light brown; S1, S11 long, thorny, robust; others shorter, smooth; Vx setae small.

Gena, ventral region of head capsule brown, with white spot in anterior region of genal mark. Labial palpus white, marked with light brown on basal segment, distal two annuli of middle segment, base of terminal segment. Mandibles amber to light brown. Antenna with light brown scape, pedicel white basally, light brown distally; flagellum light brown.

*Thorax* (Figs 2B, 10B) mostly white, with sclerotized structures light brown to brown, small patches of light brown; episternum light brown. Legs white; base of coxa brown; tibia, tarsus tinged with very light brown; tarsal claws, empodia, brown. LS light brown to brown; other setae amber to light brown.

T1: Row of three very small setae (R1) at anteromesal base of LTs not observed. Sc1 with edges brown, center white; S2Sc1 very small, immediately above S1Sc1. S1, S3 intermediate-length, robust. T2: Spiracle with lip of atrium protruding above integumental surface. Sc1, Sc2, Sc3 light brown; S2Sc3, S2 long, robust, thorny, of approximately equal size. T3: S1Sc1, S2Sc1, S1Sc2 very small; S2Sc2 absent. Raised posterior fold with row (R1) of four very long, robust, thorny setae on chalazae with ovate, light brown marks anteriorly.

*Abdomen* (Figs 10C–D) white to cream-colored, with light dusting of brown, especially on A4-A6; sides and venter of these segments also with diffuse brown tinge. LTs, LDTs white with brown chalazae; chalazae of most dorsal setae brown.

A1-A5: Dorsum with 12 SMS in two rows, with four mesal pairs equally spaced, two lateral-most pairs juxtaposed; spiracle with SSp elongate, robust, mesal to spiracle; chalazae of all SMS and SSp large, robust. A6: Anterior region with two SMS. Spiracle with very small SSp mesally. A7: Anterior region without setae or microsetae. Spiracle without apparent SSp. A8: Venter with one pair of medium-length setae posteriorly.

#### Second and third instars.

**Figure 11.**
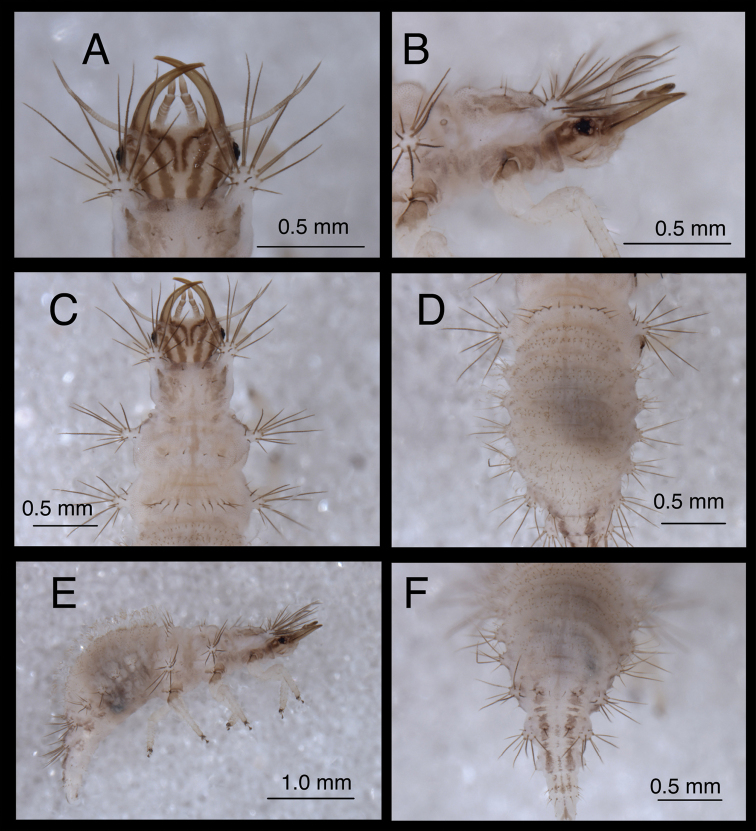
*Chrysopodes (Chrysopodes) fumosus*, second instar **A** Head, dorsal **B** Head, lateral **C** Head and thorax, dorsal **D** Abdominal segments A1 to A5, dorsal **E** Habitus, lateral **F** Abdominal segments A6 to A10, dorsal.

**Figure 12. F12:**
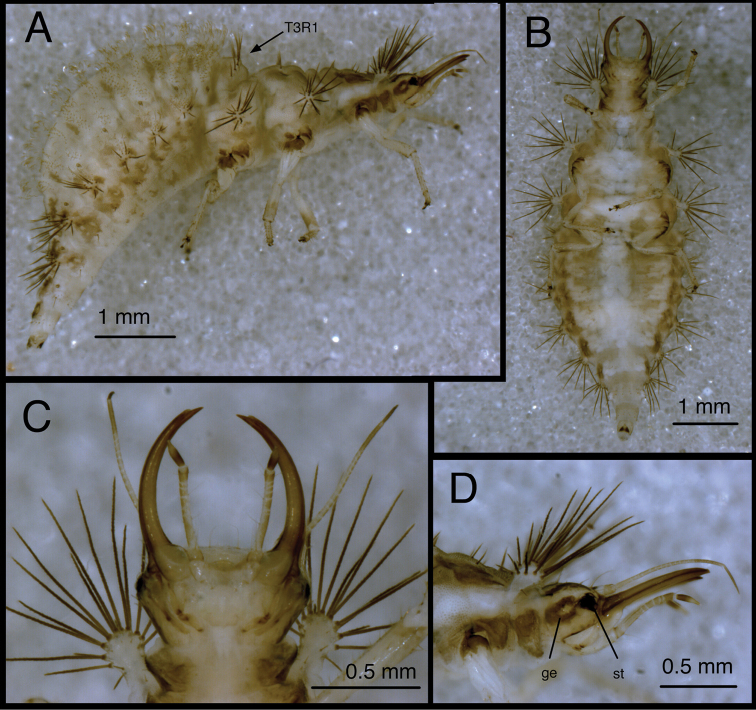
*Chrysopodes (Chrysopodes) fumosus*, third instar **A** Habitus, lateral **B** Habitus, ventral **C** Head, ventral **D** Head, lateral. *Abbreviations*: **ge** genal marking **st** stemmata **T3R1** row of long, sturdy, thorny setae on raised posterior fold of metathorax.

(Semaphoront B). *Body* (Figs 11E, 12A) length 3.1–3.4 mm (L2), 6.7–7.2 mm (L3); surface cream-colored, with light to dark brown integumental spinules, brown to dark brown dorsal, lateral markings on thorax and abdomen.

*Head* (Figs 3B, 4B, 11A-B, 12C-D) cream-colored, with dark brown markings. Epicranial marking dark brown, consisting of two narrow, elongate arms, separate from each other, both in contact with posterior margin of head; lateral arm extending from distal ~one-fifth of posterior cranial suture to upper level of eye, not touching eye, becoming broader distally; mesal arm extending from base of head, becoming confluent with postfrontal marking, which extends to inner base of scape. Postfrontal marking very dark brown, narrow throughout. Frontal marking dark brown, with each arm narrow, separate, extending from midsection of head, beyond tentorial pits to inner base of mandibles; darkness extending onto mandibles. Intermandibular marking absent. Clypeolabral region cream-colored tinged with light brown. Gena cream-colored, with large, brown marking from base of eye almost to posterior margin of cranium, with cream-colored mesal spot. Mandible, maxilla amber basally, mesally, becoming dark brown distally. Labial palpus: basal segment cream-colored with very slight tinge of brown; mesal segment ringed with light brown laterally, cream-colored mesally, with terminal subsegment brown; terminal segment dark brown basally, light brown distally. Antenna: scape and pedicel (basal, mesal sections) cream-colored, distal section of pedicel light brown, flagellum brown. Venter cream-colored, except margin of cranium, cardo with longitudinal brown marks; mentum unmarked.

All cephalic setae smooth, pointed; S1-10, S12 of medium length, S11 long; Vx setae fairly long, robust.

Head width across eyes, 0.57–0.58 mm (L2), 0.77–0.85 mm (L3); mandible length 0.51–0.57 mm (L2), 0.82–0.85 mm (L3); ratio mandible length to head width = 1.0–1.1 : 1 (L2), 0.97–1.1 :1 (L3). Tip of mandible with four teeth mesally.

Cervix cream-colored, tinged with light brown dorsally; sides with pair of broad brown patches; venter cream-colored mesally, light brown laterally.

**Figure 13.**
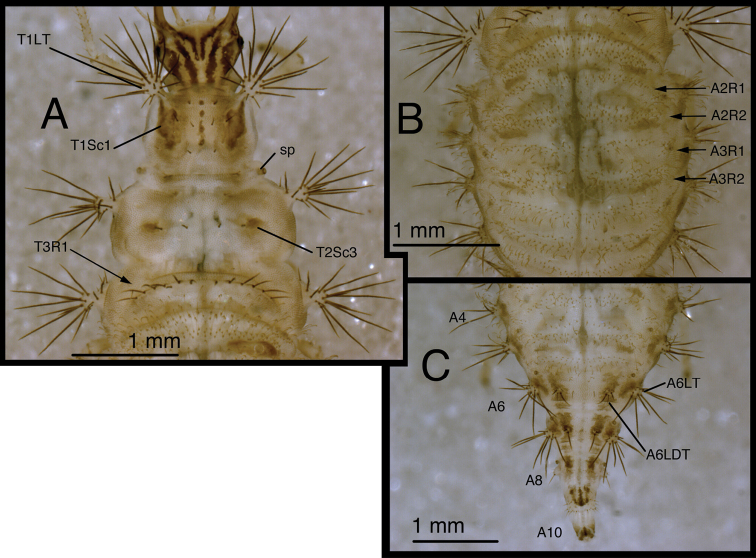
*Chrysopodes (Chrysopodes) fumosus*, third instar **A** Thorax, dorsal **B** Abdominal segments A1 to A5, dorsal **C** Abdominal segments A4 to A10, dorsal. *Abbreviations*: **A4, A6, A8, A10** abdominal segments **A2R1** double row of submedian setae (SMS) on anterior fold of second abdominal segment **A2R2** double/triple row of SMS on posterior fold of second abdominal segment **A3R1** double row of SMS on anterior fold of third abdominal segment **A3R2** double/triple row of SMS on posterior fold of third abdominal segment **A6LDT, A6LT** laterodorsal tubercle, lateral tubercle on sixth abdominal segment **sp** spiracle (on anterior subsegment of mesothorax) **T1LT** prothoracic lateral tubercle **T1Sc1** first primary prothoracic sclerite **T2Sc3** third primary mesothoracic sclerite **T3R1** row of long, sturdy, thorny setae on raised posterior fold of metathorax.

*Thorax* (Figs 3B, 4B, 11C, 12A-B, 13A) cream-colored, tinged with light brown, with sclerites, markings brown; LTs white to cream-colored, with LS brown to dark brown. Venter white to cream-colored, unmarked. Legs: coxa white, with dark brown marking on basodorsal surface; trochanter, base of femur cream-colored; tip of femur with light brown band; tibia, tarsus, tinged with light brown; empodium, claws, base of claws dark brown.

T1: LT with 14–18 (L2), 16–20 (L3) LS. Sc1 large, rhomboid, extending around posterior base of LT, darker brown laterally than mesally. Sc2 triangular, mostly brown, with small secondary sclerite above. Notum with four brown spots in longitudinal row along midline. S2, S3 thorny. T2: Anterior sclerite (Sc1) light brown; spiracles on small protuberances. Posterior subsegment with Sc2 light brown; Sc3 pronounced, brown. LT with 13–17 (L2), 16–20 (L3) LS. T3: LT with 11–15 (L2), 14–18 (L3) LS. Posterior fold with row (R1) of twelve robust, thorny setae.

*Abdomen* (Figs 11D–F, 12A–B, 13B–C) largely cream-colored, tinged with light brown, with light brown to brown markings surrounding bases of LTs; markings becoming darker posteriorly; white fat-body visible beneath midline section (especially posteriorly); robust, thorny setae dark brown; other setae light brown to amber-colored. A5 with pair of light brown spots mesal to LTs. A6, A7 each with pair of large, diffuse, dark brown marks surrounding LDTs. A8 with pair of dark brown marks mesal to spiracles. A9 with U-shaped dorsal mark containing darker longitudinal mark along midline, with pair of lighter brown marks extending onto top of LTs. A10 dark brown distally. Venter white along midsection, cream-colored to light brown laterally; areas mesal to LTs with extensive, diffuse brown marks. Midsection of A10 with pair of small, abutting, triangular dark brown marks.

A1: Dorsum with ~54–59 (L2), ~90–94 (L3) SMS in two double-triple transverse bands between spiracles. A2-A5: Dorsum with 42–86 (L2), 100–166 (L3) SMS in two broad transverse bands. LTs each with 9–13 (L2), 13–20 (L3) LS; apical four to eight LS long, robust, thorny, pointed to blunt; remaining LS less robust, smooth, hooked, in patch on dorsal surface. A6: Dorsum with transverse band of ~26 (L2), ~45 (L3) SMS across anterior of segment; midsection with two pairs of smooth setae, mesal pair hooked, lateral pair pointed. LTs with ~9 (L2), ~15 (L3) LS of various sizes. A7: Dorsum with two pairs of very short setae (S1, S2) anteriorly, between spiracles. LDTs each with one medium-length, robust, thorny, blunt to spatulate LDS, one to two shorter, thorny, robust LDS, one to three small, smooth, pointed LDS. LTs with ~8 (L2), ~11 (L3) LS of various sizes. A8: Dorsum with two to three pairs of very small setae anteriorly; two to three pairs of small setae posteromesal to spiracles; four pairs of small, posterior setae in transverse row mesal to LTs. Venter with three transverse rows of setae, each with three to four smooth, pointed setae of increasing size posteriorly. A9: Dorsum with one pair of very small setae anteriorly. Middle and posterior regions with two transverse rings of setae extending around segment; each ring with ~16–18 short to medium-length setae, several in each ring robust. A10: Dorsum with two pairs of small setae: one posterior to V-shaped anterior sclerites, one slightly anterior to terminus. Two pairs of lateral setae, robust. Venter with ~five pairs of small setae, posterior row of microsetae anterior to terminus.

#### Egg.

At oviposition, light green, with white micropyle; ovoid, 0.99 to 1.08 mm long, 0.40 to 0.41 mm wide. Stalk smooth, hyaline, 4.39 to 5.42 mm long.

#### Larval specimens examined.

Several lots, each originating from a single gravid female collected in **Brazil, Rio de Janeiro**: Conceição de Macabu, Santo Agostinho, V-21-2002, V-2-2003 (Albuquerque Lot 2002:012, Tauber Lots 2002:021, 2003:016); two field-collected L3, Conceição de Macabu, Santo Agostinho, V-2-2003.

#### Biology.

Adults and larvae of *Chrysopodes (Chrysopodes) fumosus* were collected on citrus and other fruit trees in mixed agricultural situations. The adults are very agile; they make short, fast, evasive flights, usually downward and toward the interior of the tree. In the lab, eggs were deposited separately (with isolated stalks), in small groups with no particular pattern; the stalks were sticky, but without droplets. The larvae carry pieces of woody plant material and other debris; they are agile, but not particularly fast moving.

### Chrysopodes (Chrysopodes) geayi

(Navás, 1910)

http://species-id.net/wiki/Chrysopodes_geayi

Figs 2 [Fig F3] –4 14 [Fig F15] [Fig F16] [Fig F17] –18

#### Discussion.

Among *Chrysopodes (Chrysopodes)* species, *Chrysopodes (Chrysopodes) geayi* [= *Chrysopodes (Chrysopodes) pulchellus* (Banks); see Legrand et al. 2008] are relatively large bodied. Adults have distinctive forewings: tall costal cells (especially basally); quadrate intramedian cell; sinuate subcostal, radial and radial sector veins; irregular gradate veins. Both the female and male genitalia are distinctive. Adults of this species can be identified using the key and information in Adams and Penny [1985: 423, as *Chrysopodes (Chrysopodes) pulchella*].

#### Known geographic distribution.

Brazil (Banks 1910: 152, as *Allochrysa pulchella*), French Guiana (Navás 1910), Suriname (Banks 1944, not confirmed). See Adams and Penny (1985), Adams (1985), Legrand et al. (2008).

#### Larval diagnosis.

*Chrysopodes (Chrysopodes) geayi* larvae are recognized by their relatively large size, dense setation, and unique pronotal Sc1 sclerites (having dark brown lateral and mesal bands and an unmarked, cream-colored central band). The *Chrysopodes (Chrysopodes) geayi* dorsal head markings resemble those of *Chrysopodes (Chrysopodes) fumosus* and *Chrysopodes (Chrysopodes) spinellus* (longitudinally elongate and divided, brown epicranial markings; unmarked intermandibular and clypeal regions). However, unlike as in *Chrysopodes (Chrysopodes) fumosus* [but not *Chrysopodes (Chrysopodes) spinellus*], the posterior ends of the frontal markings curve and connect with each other mesally. Semaphoront A of *Chrysopodes (Chrysopodes) geayi* is distinguished from that of *Chrysopodes (Chrysopodes) spinellus* by the presence of thorns on only two or three cranial setae (S1, S11, sometimes S4) and its dark brown thoracic and abdominal LS. The *Chrysopodes (Chrysopodes) geayi* Semaphoront B differs from *Chrysopodes (Chrysopodes) spinellus* [but not *Chrysopodes (Chrysopodes) fumosus*] by the presence of several secondary sclerites on the pronotal midline.

#### First instar.

**Figure 14.**
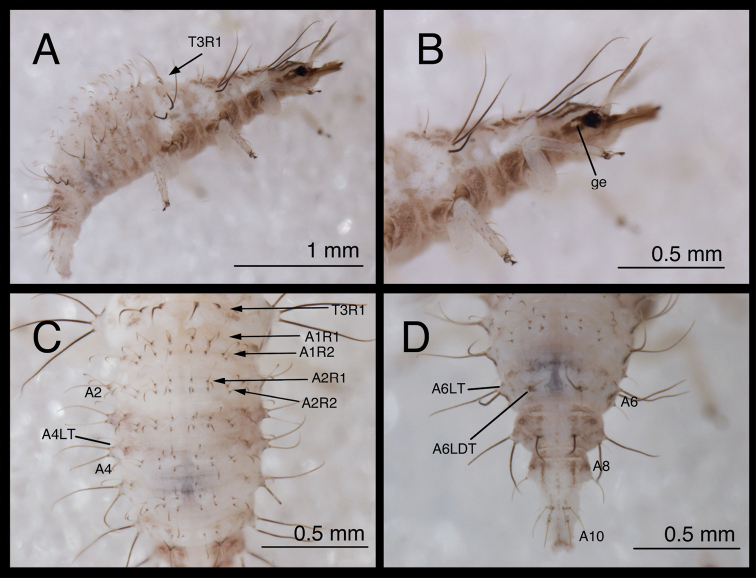
*Chrysopodes (Chrysopodes) geayi*, first instar **A** Habitus, lateral **B** Head, lateral **C** Abdominal segments A1 to A5, dorsal **D** Abdominal segments A6 to A10, dorsal. *Abbreviations*: **A2, A4, A6, A8, A10** abdominal segments **A1R1, A1R2** anterior and posterior rows of submedian setae (SMS) on first abdominal segment **A2R1, A2R2** anterior and posterior rows of SMS on second abdominal segment **A4LT** lateral tubercle on fourth abdominal segment **A6LDT, A6LT** laterodorsal tubercle, lateral tubercle on sixth abdominal segment **ge** genal marking **T3R1** row of long, sturdy, thorny setae on raised posterior fold of metathorax.

(Semaphoront A). *Body* (Fig. 14A) 2.7–3.1 mm long. Surface predominantly white to cream-colored, with some small, light brown marks, light dusting of brown, especially on sides and venter.

*Head* (Figs 2C, 14B) 0.42–0.45 mm wide; mandibles 0.37–0.39 mm long (ratio, mandible length : head width = 0.84–0.92 : 1). Cranium mostly white, with elongate, brown to dark brown markings. Epicranial marking consisting of two, unconnected, elongate, brown stripes; mesal arm contiguous with postfrontal marking, extending from base of cranium to anteromesal margin of antennal socket; lateral arm brown, especially dark basally, extending from posterolateral margin of cranial suture approximately to distal base of mandible. Postfrontal marking indistinguishable, fused with epicranial marking. Frontal marking narrow, paired, extending from midregion of cranium anteriorly to level of antennal socket, then bending laterally toward mesal margin of mandibular base; mesal ends of marking curving inward, fusing narrowly at tip. Intermandibular, clypeal area white. Cranial setae amber to brown; S1, S11 long, thorny; S4 intermediate-length, smooth or thorny; others short, smooth.

Gena brown with small central white spot, ventral region of head capsule white to cream-colored, with ventral margin of head capsule brown. Labial palpus white, with tinge of light brown on basal segment, lateral side of middle segment, darker brown on distal annulation, terminal segment. Mandibles amber to light brown, with dark brown basally. Antenna with light brown scape, pedicel light brown basally, brown distally; flagellum brown. Cervix with pair of light brown sublateral spots.

*Thorax* (Figs 2C, 14B) mostly white, with small to large patches of brown forming pair of submedian longitudinal, brown bands; sclerotized structures mostly light brown; pleural region with distinct brown band running through episterna, epimera, membranes between; episternum, epimeron dark brown. Legs white; base of coxa dark brown, tibia, tarsus tinged with very light brown; tarsal claws, empodia, brown. LS dark brown; other setae amber to dark brown.

T1: Row of three very small setae (R1) at anteromesal base of LTs present. Sc1 with distal section light brown, center white; S2Sc1 very small, immediately above S1Sc1. S1 intermediate-length; S3 intermediate-length to short, thorny. T2: Spiracle with lip of atrium protruding slightly beyond level of integumental protuberance. Sc1, Sc2, Sc3 light brown; S2Sc3, S2 small to intermediate length, thorny, of approximately equal size. T3: S1Sc1, S2Sc1, S1Sc2 very small; S2Sc2 absent. S1, S2 absent. Raised posterior fold with row (R1) of four very long, robust, thorny, pointed setae on chalazae with ovate, light brown marks anteriorly.

*Abdomen* (Figs 14A, 14C–D) white, with light dusting of brown, especially on sides and venter of A2-A6, dorsum of A7, A8. LTs, LDTs marked with brown; most dorsal chalazae brown to dark brown.

A1-A5: Dorsum with 12 SMS in two rows, with lateral-most two pairs juxtaposed. Spiracle with SSp elongate, robust, mesal to spiracle. A6: Anterior region with two SMS. Spiracle with SSp mesally. A7: Anterior region without setae or microsetae. Spiracle anterior to LT, without apparent SSp. A8: Venter with two pairs of medium-length setae posteriorly.

#### Second and third instars.

**Figure 15. F15:**
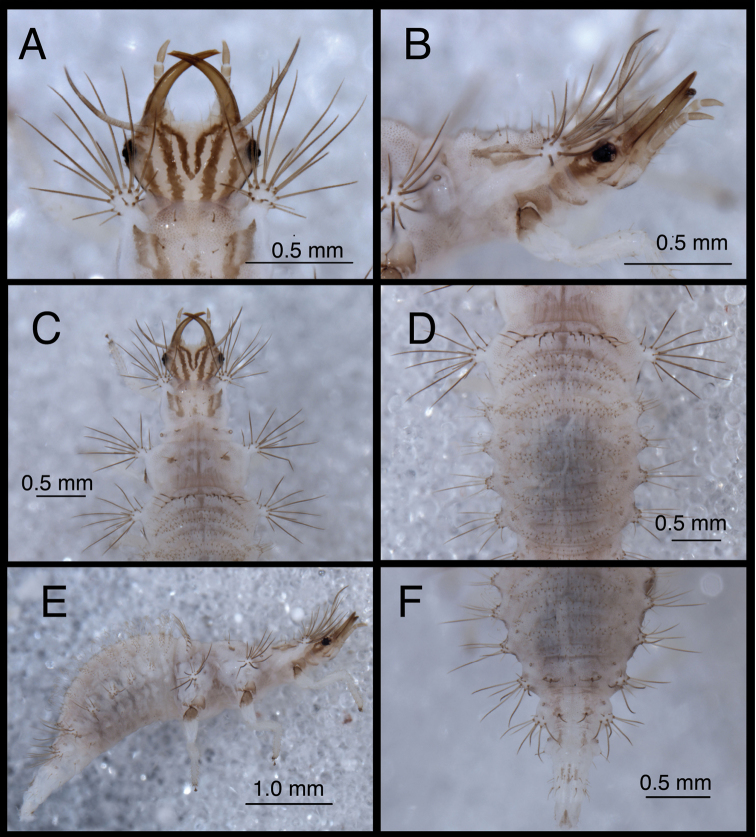
*Chrysopodes (Chrysopodes) geayi*, second instar **A** Head, dorsal **B** Head, lateral **C** Head and thorax, dorsal **D** Abdominal segments A1 to A5, dorsal **E** Habitus, lateral **F** Abdominal segments A6 to A10, dorsal.

**Figure 16. F16:**
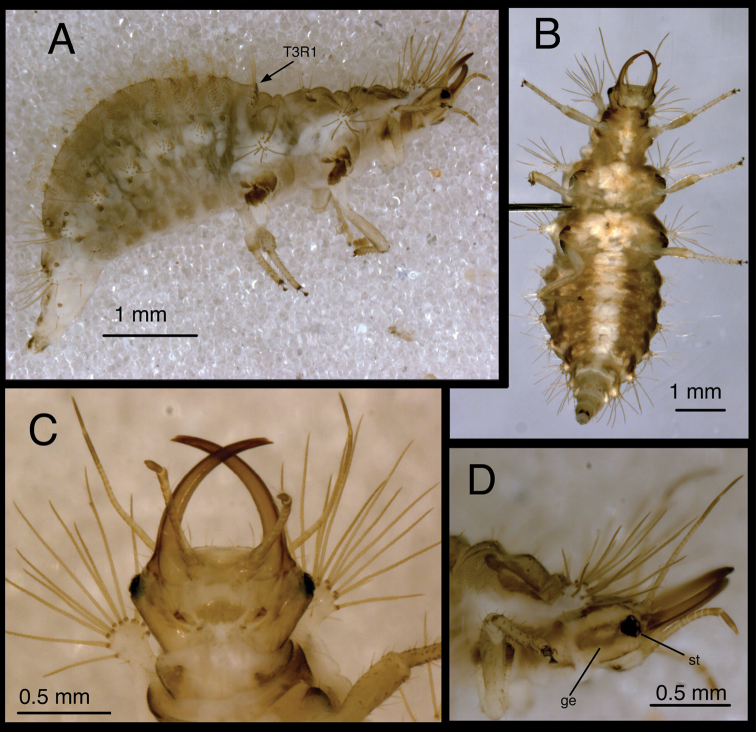
*Chrysopodes (Chrysopodes) geayi*, third instar **A** Habitus, lateral **B** Habitus, ventral **C** Head, ventral **D** Head, lateral. *Abbreviations*: **ge** genal marking **st** stemmata **T3R1** row of long, sturdy, thorny setae on raised posterior fold of metathorax.

(Semaphoront B). *Body* (Figs 15E, 16A–B) length 4.6–5.1 mm (L2), 7.5–7.9 mm (L3); surface white to cream-colored, with light to dark brown integumental spinules especially dense, dark on pronotum; primary pronotal, mesonotal sclerites brown to dark brown, other dorsal marks brown; sclerites around coxae, base of coxae dark brown, abdomen (lateral) with brownish hue interrupted by white lateral stripe through abdominal lateral tubercles.

*Head* (Figs 3C, 4C, 15A–B, 16C–D) cream-colored, with brown to dark brown markings. Epicranial marking brown, with two elongate arms, separate from each other, both in contact with posterior margin of head; lateral arm extending from distolateral margin of cranium to upper level of eye, touching eye, tapering distally; mesal arm extending from base of head, becoming confluent with postfrontal marking, which extends to inner margin of scape. Postfrontal marking dark brown, narrow throughout. Frontal marking dark brown, with each arm narrow, separate (except at basal tip), extending from midsection of head, beyond tentorial pit to inner base of mandible; base of each arm tapering, turning mesally, contacting tip of other arm. Intermandibular marking absent. Clypeolabral region cream-colored, tinged with light brown. Gena cream-colored, with large, brown marking from base of eye to posterior margin of cranium, with small, cream-colored mesal spot. Mandible, maxilla amber basally, mesally, dark brown laterally, distally. Labial palpus: basal segment cream-colored with very slight tinge of brown; mesal segment ringed with brown laterally, cream-colored mesally, with terminal subsegment brown; terminal segment brown. Antenna: scape and pedicel (basal, mesal sections) light brown, distal one-fifth of pedicel, entire flagellum, darker brown. Venter cream-colored, with large, white central area; margin of cranium, cardo with dark brown longitudinal marks; mentum with large, brown spot basally.

Cephalic seta S1 moderately long, thorny, S2-S12 smooth, S11 long; Vx setae relatively long.

Head width across eyes, 0.64–0.66 mm (L2), 0.98–1.05 mm (L3); mandible length, 0.51–0.58 mm (L2), 0.91–0.98 mm (L3); ratio mandible length to head width = 0.82–0.87 : 1 (L2), 0.93–0.99 : 1 (L3). Tip of mandible with four teeth mesally.

Cervix: dorsum cream-colored, tinged with light brown; sides with pair of broad brown patches; venter brown laterally, becoming cream-colored mesally.

**Figure 17. F17:**
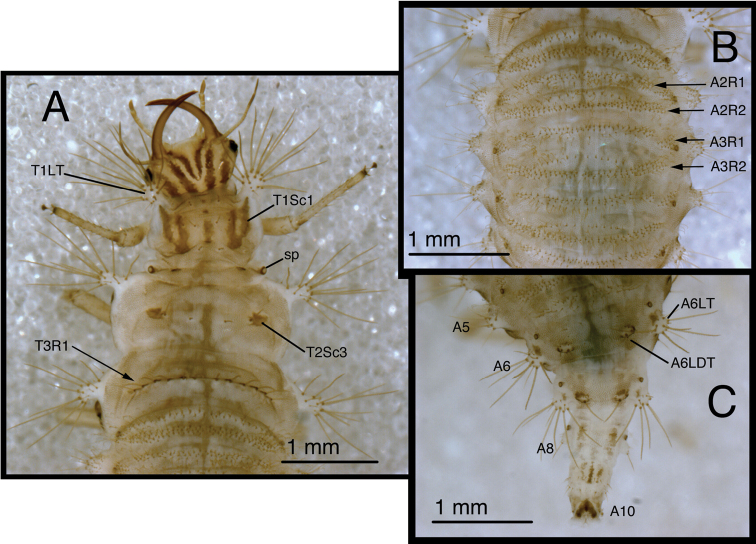
*Chrysopodes (Chrysopodes) geayi*, third instar **A** Thorax, dorsal **B** Abdominal segments A1 to A4, dorsal **C** Abdominal segments A5 to A10, dorsal. *Abbreviations*: **A5, A6, A8, A10** abdominal segments **A2R1** double row of submedian setae (SMS) on anterior fold of second abdominal segment **A2R2** double/triple row of SMS on posterior fold of second abdominal segment **A3R1** double row of SMS on anterior fold of third abdominal segment **A3R2** double/triple row of SMS on posterior fold of third abdominal segment **A6LDT**, **A6LT** laterodorsal tubercle, lateral tubercle on sixth abdominal segment **sp** spiracle (on anterior subsegment of mesothorax) **T1LT** prothoracic lateral tubercle **T1Sc1** first primary prothoracic sclerite **T2Sc3** third primary mesothoracic sclerite **T3R1** row of long, sturdy, thorny setae on raised posterior fold of metathorax.

*Thorax* (Figs 3C, 4C, 15C, 15E, 16A–B, 17A) white to cream-colored; dorsum lightly tinged with brown, the darkness of which depends on density, color of integumental spinules; with sclerites, markings brown; LTs white, with LS amber to light brown. Venter white to cream-colored, with light brown tinge laterally where spinules extend toward ventral surface, without other marks. Legs: coxa white, with dark brown on dorsal surface; trochanter cream-colored to white, base of femur cream-colored, becoming brownish distally; tibia white to tinged with very light brown, with light brown setae; tarsus very light brown with dark tip; empodium brown; claws amber.

T1: LT with 15–19 (L2), 18–22 (L3) LS. Sc1 with elongate brown marks laterally, mesally, cream-colored streak centrally; two heavily sclerotized rods extending from base of sclerite, mesal one stretching along mesal margin of Sc1 to mesal base of LT, lateral one forked distally, with mesal fork extending onto posterolateral margin of LT, lateral fork extending laterally below posterior base of LT. Sc2 triangular, included in elongate mesal brown mark, with two small, brown, secondary sclerites anteriorly. S2, S3 thorny. T2: Anterior sclerite (Sc1) brown; spiracles on small protuberances. Posterior subsegment with Sc2 light brown; Sc3 pronounced, brown. LT with 15–19 (L2, L3) LS. T3: LT with 14–18 (L2, L3) LS. Posterior fold with row (R1) of twelve robust, thorny setae.

*Abdomen* (Figs 15D-F, 16A-B, 17B-C) white to cream-colored, with light brown chalazae and setae; white fat-body visible beneath integument; setae mostly amber-colored. A6, A7 each with pair of large, dark brown marks dorsal to LTs; LDTs white with dark brown chalazae, brown marks anterior and posterior to LDTs. A8 with pair of dark brown marks mesal to spiracles. A9 with three elongate brown marks dorsally. A10 with inverted V-shaped, dark brown dorsal mark; light brown laterally. Venter white to cream-colored, without marks, except with some light brown pigmentation ventrolaterally; tip of A10 with pair of small, triangular dark brown marks.

A1: Dorsum with 62–76 (L2), ~ 156–188 (L3) SMS in two double-triple transverse bands between spiracles. A2-A5: Dorsum with 62–114 (L2), 176–268 (L3) SMS in two broad transverse bands. LTs each with 9–14 (L2), 12–34 (L3) LS: four to eight long, robust, thorny, pointed to spatulate LS on distal surface; remaining LS less robust, smooth, hooked, in patch on dorsal surface. A6: Dorsum with transverse band of 22–32 (L2), 42–56 (L3) SMS across anterior of segment; midsection with two pairs of smooth setae, mesal pair hooked, lateral pair pointed. LT with 8–10 (L2), ~11–14 (L3) LS of various sizes. A7: Dorsum with three pairs of very short setae anteriorly, between spiracles. LT with ~8–10 (L2), 11–14 (L3) LS of various sizes. A8: Dorsum with two pairs of very small setae between spiracles; four pairs of small setae in transverse row between LTs. Venter with two transverse rows of setae, each with three to four smooth, small, pointed setae. A9: Dorsum with one pair of very small setae anteriorly. Middle and posterior regions with two transverse rings of setae extending around segment; each ring with ~14–16 short to medium-length setae, several in each ring robust. A10: Dorsum with two pairs of small setae: one posterior to V-shaped anterior sclerites, one slightly anterior to terminus. Two pairs of lateral setae, robust. Venter with ~five pairs of small setae, posterior row of microsetae anterior to terminus.

#### Egg.

Green, ovoid, 0.95–1.00 mm long; 0.43–0.45 mm wide; stalk hyaline, 10.7–12.6 mm long.

#### Larval specimens examined.

Several lots, each originating from a single gravid female collected in **Brazil, Rio de Janeiro**: Campos dos Goytacazes, Parque Estadual do Desengano, Babilônia III-27-2001, X-26-2003 (Tauber Lot 2001:003, Albuquerque Lot 2003:018); Campos dos Goytacazes, near Parque Estadual do Desengano, Fazenda Boa Vista, V-16-2002 (Tauber Lot 2002:016); Campos dos Goytacazes, Distrito de Morangaba, Fazenda São Julião, X-18-2005 (Tauber Lot 2005:034). Two field-collected specimens from RJ, all collected by Albuquerque, Tauber and Tauber: an L3 collected from Santo Antônio do Imbé, Parque Estadual do Desengano on III-31-1999, and an L3 from the Babilônia site, collected on V-4-2003.

#### Biology.

**Figure 18.**
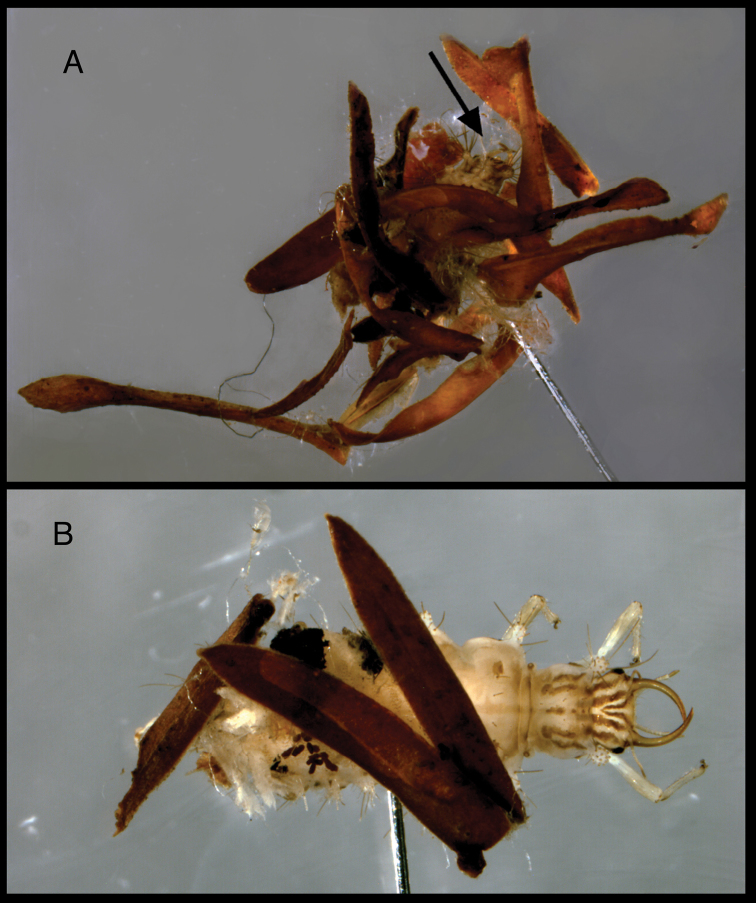
*Chrysopodes (Chrysopodes) geayi*, field-collected L3 with dorsal trash packet **A** Campos dos Goytacazes, Parque Estadual do Desengano, Babilônia, V-4-2003, Albuquerque, Tauber and Tauber (TRC) [Arrow indicates dorsum of head] **B** Santo Antônio do Imbé, Parque Estadual do Desengano, III-31-1999, Albuquerque, Tauber and Tauber (TRC).

Eggs of *Chrysopodes (Chrysopodes) geayi* are deposited separately, with isolated stalks, in no particular pattern. Adults were collected in disturbed, forested areas of coastal Brazil. Occasionally, we encountered fairly substantial numbers in moist habitats near streams. The two larvae (L2, L3) from small trees in the field at Babilônia and Santo Antônio do Imbé (see data above) were both carrying relatively sparse coverings of plant material (brownish leaflets or bracts) and fibers loosely attached to the dorsal setae. On the two specimens from different localities, the leaflets or bracts appeared to be from the same or similar type of plant (Fig. 18).

### Chrysopodes (Chrysopodes) lineafrons

Adams & Penny, 1987

http://species-id.net/wiki/Chrysopodes_lineafrons

Figs 2 [Fig F3] –4 19 [Fig F20] [Fig F21] –22

#### Discussion.

*Chrysopodes (Chrysopodes) lineafrons* occurs widely throughout South America where it frequently has been reported from cropping systems, especially tropical fruit orchards (Adams and Penny 1985, González Olazo et al. 1999, Freitas and Penny 2001, Silva et al. 2007). It is a relatively small-bodied species that can be recognized by its facial markings, parallel and dark gradate veins, and distinctive genitalia (male and female). Adults can be identified using the keys in Adams and Penny (1985) and Freitas and Penny (2001).

#### Known geographic distribution.

Argentina (González Olazo et al. 1999); Brazil (Adams and Penny 1985, Freitas and Penny 2001, Silva et al. 2007).

#### Larval diagnosis.

The dorsal head markings of *Chrysopodes (Chrysopodes) lineafrons* larvae are similar to those of *Chrysopodes (Chrysopodes) divisus* (Figs 2–4). However, *Chrysopodes (Chrysopodes) lineafrons* (Semaphoront A) can be differentiated from *Chrysopodes (Chrysopodes) divisus* by the smaller number of robust, thorny setae on the posterior fold (R1) of the metathorax (n = 4) and the smaller number of smooth, hooked SMS on abdominal segments A1-A5 (n = 16). A dense coating of spinules on the dorsal integument gives the *Chrysopodes (Chrysopodes) lineafrons* larval body (Semaphoront B) a distinctive dark brown hue (most notable on the thorax and abdominal segments A1-A6, including the pleural and ventral regions). Other distinguishing characteristics of *Chrysopodes (Chrysopodes) lineafrons* Semaphoront B are the thorny cranial setae S1, S4, S6, S11, the large, dark brown pronotal plates (Sc1), and the presence of secondary sclerites on the pronotal midline.

#### First instar.

**Figure 19.**
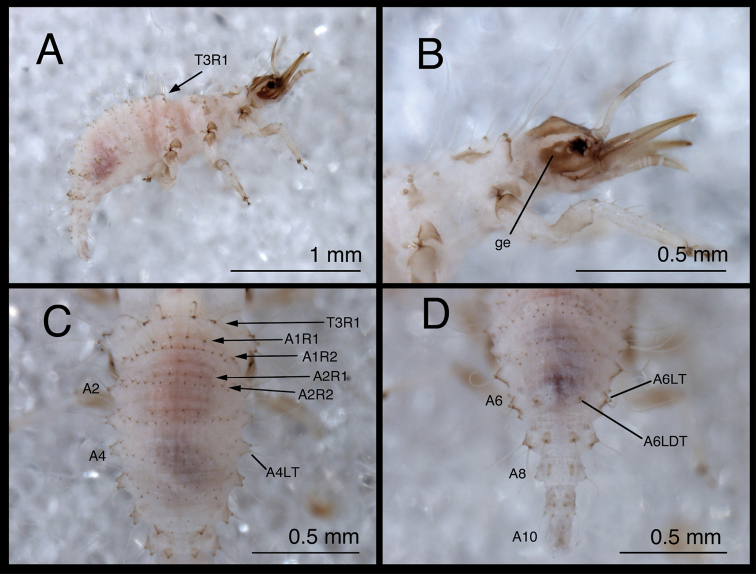
*Chrysopodes (Chrysopodes) lineafrons*, first instar **A** Habitus, lateral **B** Head, lateral **C** Abdominal segments A1 to A5, dorsal **D** Abdominal segments A6 to A10, dorsal. *Abbreviations*: **A2, A4, A6, A8, A10** abdominal segments **A1R1, A1R2** anterior and posterior rows of submedian setae (SMS) on first abdominal segment **A2R1, A2R2** anterior and posterior rows of SMS on second abdominal segment **A4LT** lateral tubercle on fourth abdominal segment **A6LDT, A6LT** laterodorsal tubercle, lateral tubercle on sixth abdominal segment **ge** genal marking **T3R1** row of long, sturdy, thorny setae on raised posterior fold of metathorax.

(Semaphoront A). *Body* (Fig. 19A) 2.5–2.6 mm long; surface predominantly white to cream-colored, with some, small, light brown to brown markings.

*Head* (Figs 2D, 19B) 0.40–0.41 mm wide; mandibles 0.35–0.37 mm long (ratio, mandible length : head width = 0.85–0.90 : 1). Cranium mostly brown, with elongate, white to cream-colored area posteromesally extending from margin of cranium anteriorly to base of frontal markings, pair of white to cream-colored areas between lateral arm of epicranial marking and postfrontal marking. Epicranial marking light brown mesally, darker brown laterally, with mesal and lateral arms fused posteriorly; lateral arm extending around antennal sockets, above eyes; mesal arm fused with postfrontal marking. Postfrontal marking diffuse, darker brown than most of epicranial marking, contiguous basally with distal margin of epicranial marking (mesal arm), extending anteriorly almost to mesal margin of antennal base. Frontal markings thin, dark brown, paired but fused mesally, extending laterally across anterior of head to middle of mandibular base, merged with brown intermandibular coloration. Cranial setae light amber; S1, S4, S6, S11 thorny; S1, S11 long, others shorter; Vx setae very small.

Gena, ventral margin of head capsule brown, with small clear patch within genal mark. Labial palpus tinged with light brown, slightly darker distally. Mandibles light brown. Antenna with light brown scape, pedicel white basally, brown distally; flagellum brown.

*Thorax* (Figs 2D, 19A) mostly cream to white, with sclerotized structures light brown to brown; episternum brown. Legs white, with base of coxa brown, femur (especially distal half) tinged with brown, dorsum of tibia tinged with light brown; tarsal claws, empodia, brown. LS light amber; other setae cream to light amber.

T1: Row of three small setae (R1) near base of LT not observed. Sc1 brown throughout, but slightly lighter mesally; S2Sc1 small, immediately above S1Sc1. S1 long; S3 intermediate-length, slightly thorny. T2: Spiracle with lip of atrium raised above integumental surface. Sc1, Sc2 transparent: Sc3 marked with light brown; S2Sc3 variable, from medium-length to long, S2 smaller than S2Sc3. T3: S1Sc1 present, S2Sc1 (sometimes absent), S1Sc2 very small. S2Sc2 absent. Raised posterior fold with row (R1) of four long, thorny, pointed setae on chalazae with ovate, light brown marks anteriorly.

*Abdomen* (Figs 19C–D) white to cream-colored, with LTs, LDTs tinged with light brown, chalazae of most dorsal setae brown.

A1-A2: Dorsum with four to six SMS in anterior row, with 12 to 16 SMS in posterior row. Spiracles far lateral to anterior row, with SSp mesally. A3-A5: Anterior and posterior rows of SMS largely coalesced, with four to six SMS in anterior row, ten to 14 SMS in curved, posterior row. Spiracle with SSp near anteromesal margin. A6: Anterior region with row of two to six SMS, pair of small, straight setae lateral to SMS. Spiracle with pair of small SSp mesally. A7: Anterior region without setae or microsetae. Spiracle with SSp mesally. A8: Venter with two pairs of medium-length setae posteriorly, one pair of short setae anteriorly.

#### Second and third instars.

**Figure 20. F20:**
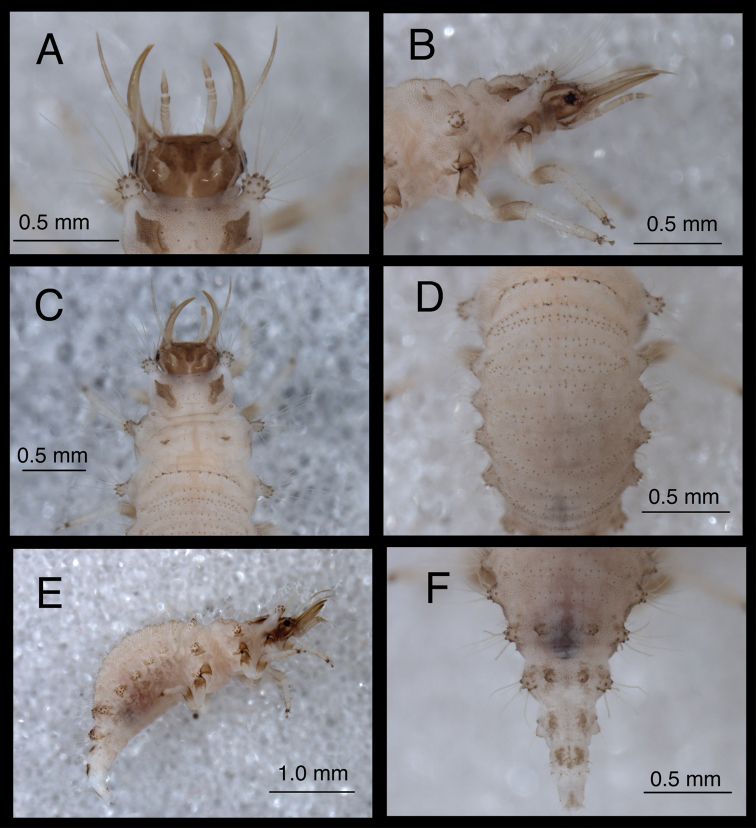
*Chrysopodes (Chrysopodes) lineafrons*, second instar **A** Head, dorsal **B** Head, lateral **C** Head and thorax, dorsal **D** Abdominal segments A1 to A5, dorsal **E** Habitus, lateral **F** Abdominal segments A6 to A10, dorsal.

**Figure 21. F21:**
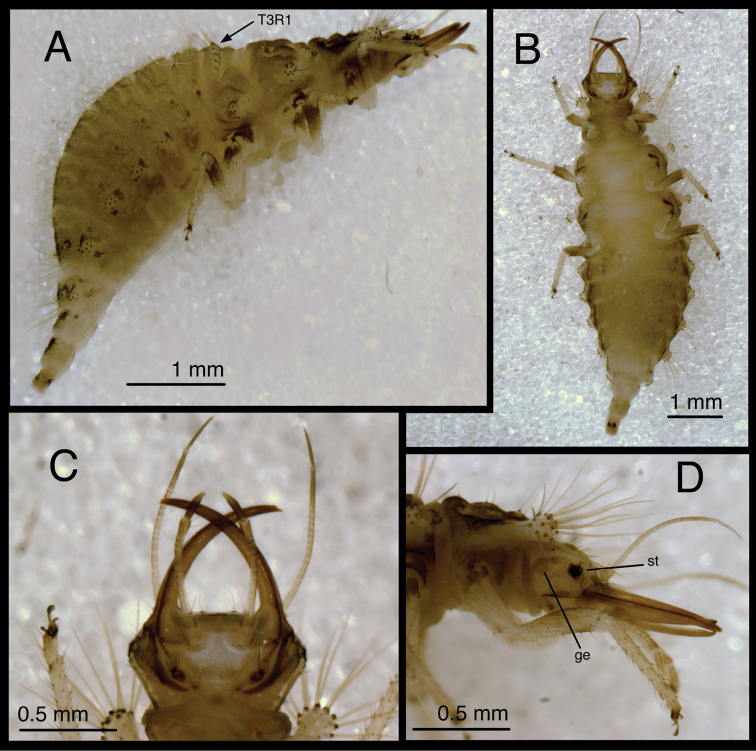
*Chrysopodes (Chrysopodes) lineafrons*, third instar **A** Habitus, lateral **B** Habitus, ventral **C** Head, ventral **D** Head, lateral. *Abbreviations*: **ge** genal marking **st** stemmata **T3R1** row of long, sturdy, thorny setae on raised posterior fold of metathorax.

(Semaphoront B). *Body* (Figs 20E, 21A–B) length 3.3–3.9 mm (L2), 5.8–6.8 mm (L3); surface white to cream-colored, with dense brown integumental spinules throughout, especially dense, dark on pronotum, mesonotum; primary pronotal, mesonotal sclerites brown to dark brown; dark brown marks anterior and posterior to lateral tubercles; sclerites anterior to coxae dark brown, lateral section of abdomen mostly light brown to brown.

*Head* (Figs 3D, 4D, 20A–B, 21C–D) dorsum cream-colored, with brown to dark brown markings. Epicranial marking brown, with mesal and lateral arms in broad contact mesally; both arms in contact with posterior margin of head; lateral arm extending from distolateral margin of cranium to upper level of eye, distal part tapering , broken, almost surrounding dorsal margin of eye; mesal arm extending from base of head, almost confluent with postfrontal marking. Postfrontal marking dark brown, narrow throughout, extending toward inner margin of scape. Frontal marking dark brown, basal arms confluent with each other, forming broad central mark, extending from midsection of head, beyond tentorial pits to intermandibular marking; base of each arm rounded. Intermandibular marking dark brown, broad mesally, tapering laterally, at base of mandibles. Clypeolabral region cream to white. Gena cream-colored, with large, brown, forked marking near base of cranium, with tips of fork reaching approximately 3/4th distance to eye. Mandible, maxilla amber basally, mesally, dark brown laterally, distally. Labial palpus: basal section cream-colored with very slight tinge of brown laterally; mesal segment tinged with brown laterally, cream-colored mesally, with terminal subsegment brown; terminal segment brown. Antenna: scape amber; basal section of flagellum cream-colored with slight tinge of brown; distal 1/4th of pedicel, flagellum, darker brown. Venter amber, with large, white central area; margin of cranium, cardo dark brown; mentum with rectangular, brown spot basally.

All cephalic setae present; S1, S11 long; S2-S10, S12 medium length to short; S1 thorny; S4, S6, S11 lightly thorny (thorniness difficult to see except under high magnification, especially on L2); other setae smooth. Vx setae relatively short. Anterior margin of head straight, with angled lateral margins; mesal pair of anterior setae medium-length, lateral two pairs short or very short.

Head width across eyes, 0.55–0.61 mm (L2), 0.91–0.96 mm (L3); mandible length, 0.49–0.55 mm (L2), 0.96–1.00 mm (L3); ratio mandible length to head width = 0.87–0.93 (L2), 1.02–1.07 (L3). Tip of mandible with six teeth mesally.

Cervix brownish, with dense covering of spinules; sides with pair of broad brown patches; venter brown throughout, darker laterally.

**Figure 22.**
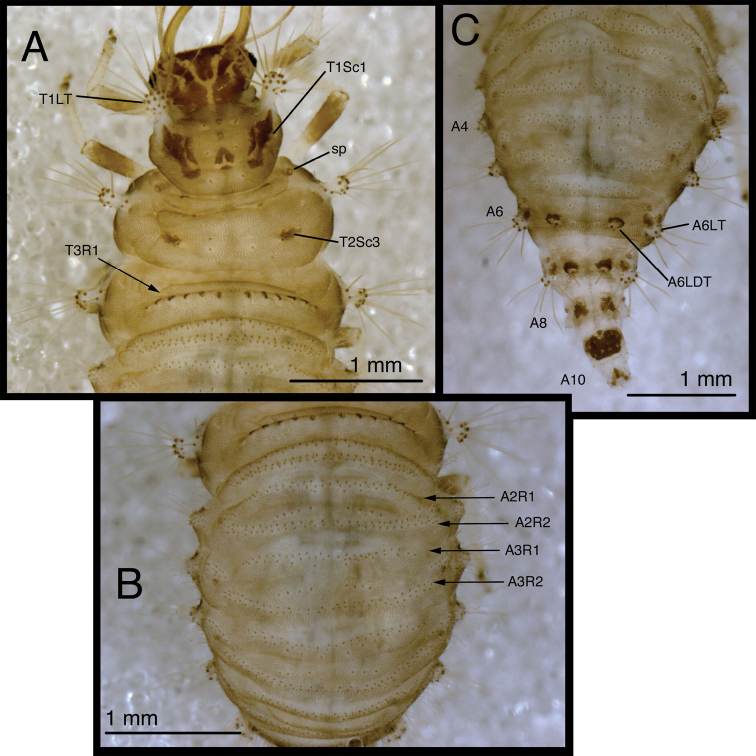
*Chrysopodes (Chrysopodes) lineafrons*, third instar **A** Thorax, dorsal **B** Abdominal segments A1 to A5, dorsal **C** Abdominal segments A4 to A10, dorsal. *Abbreviations*: **A4, A6, A8, A10** abdominal segments **A2R1** double row of submedian setae (SMS) on anterior fold of second abdominal segment **A2R2** double/triple row of SMS on posterior fold of second abdominal segment **A3R1** double row of SMS on anterior fold of third abdominal segment **A3R2** double/triple row of SMS on posterior fold of third abdominal segment **A6LDT, A6LT** laterodorsal tubercle, lateral tubercle on sixth abdominal segment **sp** spiracle (on anterior subsegment of mesothorax) **T1LT** prothoracic lateral tubercle **T1Sc1** first primary prothoracic sclerite **T2Sc3** third primary mesothoracic sclerite **T3R1** row of long, sturdy, thorny setae on raised posterior fold of metathorax.

*Thorax* (Figs 3D, 4D, 20C, 20E, 21A–B, 22A) brown dorsally, laterally, ventrolaterally, tinged by dense covering of brown spinules; sclerites, chalazae dark brown; LTs white, with LS white to light brown; chalazae cream-colored. Venter white to cream-colored mesally, with tinge of brown anteriorly, laterally, where spinules extend to ventral surface, without marks. Pleural region with small brown marks near the base of LTs. Legs: coxa white, with dark brown on dorsal surface; trochanter cream-colored to white, femur brown basally, dark brown distally; tibia white to tinged with very light brown, with light brown setae; tarsus tinged with very light brown; empodium, base brown; claws amber. Episternum with large brown mark; epimeron with small brown mark(s).

T1: LT with 15–19 (L2), 18–24 (L3) LS. Sc1 extending around posterior base of LT, dark brown laterally, mesally, with cream-colored to light brown, elongate streak with broad base. Three sclerites on midline: Sc2 triangular, cream-colored, with two brown spots anteriorly, mesal brown spot posteriorly, two smaller, light brown sclerites anterior to Sc2 [not all apparent on L2]. S2, S3 thorny. T2: Anterior sclerite (Sc1) brown; spiracle on small protuberance. Posterior subsegment with Sc2 transparent; Sc3 pronounced, dark brown. LT with 12–16 (L2), 14–18 (L3) thorny, robust LS, most long, several basal ones shorter. T3: LT with 12–16 LS (L2, L3). Posterior fold with 12–13 robust, thorny setae.

*Abdomen* (Figs 20D–F, 21A–B, 22B–C) dorsum light brown to brown throughout, LTs of A2-A5 with dark brown anterior spots, sometimes posterior spots; LS white to amber, with dark brown chalazae; chalazae of dorsal setae dark brown. A6, A7 each with pair of large, dark brown marks dorsal to LTs, pair ventral to LTs; dark brown marks surrounding anterior lateral margins of LDTs; LDTs white with dark brown chalazae; SMS amber-colored. A8 with pair of dark brown marks mesal to spiracles. A9 with dorsum almost entirely dark brown. A10 with dark brown, inverted V-shaped mark; light brown laterally. Sides of A2-A6 with large, diffuse, brown patch below each LT; venter lightly tinged with brown, light brown mesally, darker brown laterally; A7-A8 mostly white ventrally; A9 light brown ventrally; venter of A10 with pair of small, contiguous, triangular dark brown marks.

A1: Dorsum with 40–76 (L2), ~128–150 (L3) SMS in two double-triple transverse bands between spiracles; bands not extending laterally beyond spiracles. A2-A5: Dorsum with 41–102 (L2), 119–173 (L3) SMS in two broad transverse bands. LT with 10–15 (L2), 18–30 (L3) LS: five to seven robust, thorny, blunt LS of various lengths, on distal surface; remaining LS less robust, smooth, hooked in patch on dorsal surface. A6: Dorsum with transverse band of 16–28 (L2), 28–38 (L3) SMS across anterior of segment; midsection with two pairs of smooth setae, mesal pair hooked, long, lateral pair pointed, shorter. LT with 9–11 (L2), 11–17 (L3) robust, thorny, blunt LS of various sizes. A7: Dorsum with two pairs of short setae between spiracles. LT with 8–10 (L2), 10–13 (L3) LS of various sizes. Venter with setal number and size variable, usually with two pairs of setae anteriorly (both small, smooth), three pairs of sublateral setae posteriorly (two lateral-most pairs robust, thorny, mesal pair small, smooth). A8: Dorsum with two pairs of small setae between spiracles; four pairs of small setae in transverse row between LTs. Venter with scattered, small setae anteriorly, two robust, thorny setae on small chalazae between LTs. A9: Dorsum with one pair of very small setae anteriorly. Middle and posterior regions with two transverse rings of setae extending around segment; each ring with ~14–16 setae of various sizes, several in each ring robust. A10: Dorsum with one pair of setae anteriorly, two pairs mesally, patch of several setae distally; one pair of small setae posterior to V-shaped anterior sclerites. Lateral region with two pairs of robust setae, two to three pairs of smaller setae. Venter with ~two pairs of robust setae, five pairs of small setae.

#### Egg.

At oviposition, green, with white micropyle; ovoid, 0.92 to 0.99 mm long, 0.40 to 0.44 mm wide. Stalk smooth, hyaline, 3.1 to 6.2 mm long.

#### Larval specimens examined.

Several lots, each originating from a single gravid female collected in **Brazil, Bahia**: Cruz das Almas, VI-19-96 (Tauber Lot 96:020B). **Rio de Janeiro:** Campos dos Goytacazes, Estação Experimental Pesagro, VI-20-2006 (Albuquerque Lot 2006:08).

#### Biology.

Adults of *Chrysopodes (Chrysopodes) lineafrons* are commonly found in citrus and other orchards (see summary in Silva et al. 2007).

In the lab, eggs were deposited separately (with isolated stalks), in small groups with no particular pattern. During the first 24 hours of oviposition, the eggs were bright green, with dark green blotches. On the second day, they began to develop a bluish brown tone, with brownish mottling; by the third day the eggs were greyish blue to pinkish, with brown mottling. At 24 ± 1°C, hatching occurred within six days (n = 12).

Larvae of *Chrysopodes (Chrysopodes) lineafrons* carry dense packets of woody plant material and other dry debris; they exhibit a side-to-side rocking motion. Development of the various stages (population from the state of Bahia: Cruz das Almas, 24±1°C, n = 14) required: L1, 4–5 days; L2, 3 days; L3, 3–4 days; cocoon, 15 days; complete development from oviposition to adult emergence, 32 days. These data coincide well with the results from extensive rearings of *Chrysopodes (Chrysopodes) lineafrons* from the state of Rio de Janeiro (Campos dos Goytacazes) (see Silva et al. 2007).

Experimental life history studies of *Chrysopodes (Chrysopodes) lineafrons* in the laboratory and the field (southeastern Brazil) indicate: that the species can undergo development and reproduction all-year-round without interruption or dormancy; that during this time up to eight generations can be produced; and that temperature conditions play an important role in determining the rates of reproduction and development both in the lab and in the field (Silva et al. 2007). The species is considered to have excellent potential for mass rearing and for use in the biological control of pests in fruit orchards (Silva et al. 2007).

### Chrysopodes (Chrysopodes) spinellus

Adams & Penny, 1987

http://species-id.net/wiki/Chrysopodes_spinellus

Figs 2 [Fig F3] –4 , 23 [Fig F24] [Fig F25] –26

#### Discussion.

*Chrysopodes (Chrysopodes) spinellus* was described from the Amazon region (Adams and Penny 1985); since then, it has not received particular attention. However, we, and others (e.g., Freitas and Penny 2001) have collected it in Brazilian agricultural habitats. We suspect that it is one of the more widespread and common species of *Chrysopodes (Chrysopodes)* in Brazilian agricultural settings.

Although the female and male genitalia of *Chrysopodes (Chrysopodes) spinellus* are distinctive, both sexes show considerable variation, and the species is not easily distinguished from other *Chrysopodes (Chrysopodes)* species. The species will be dealt with in an up-coming revision of the subgenus *Chrysopodes* (C. A. Tauber, in preparation). Meanwhile, the keys and information in Adams and Penny (1985) and Freitas and Penny (2001) are helpful for identification.

#### Known geographic distribution.

Brazil (Adams and Penny 1985, Freitas and Penny 2001).

*Chrysopodes (Chrysopodes) spinellus* was reported from Argentina (with larval description) (González Olazo and Heredia 2010); however, the species identification in that report appears to be in error. The larva (L3) that was illustrated had a darkened head like both *Chrysopodes (Chrysopodes) divisus* and *Chrysopodes (Chrysopodes) lineafrons*, and it lacked the longitudinally elongate, separate mesal and lateral epicranial markings of *Chrysopodes (Chrysopodes) spinellus*. The illustrations more closely resemble *Chrysopodes (Chrysopodes) divisus* than *Chrysopodes (Chrysopodes) lineafrons*.

#### Larval diagnosis.

Like the larvae of *Chrysopodes (Chrysopodes) geayi* and *Chrysopodes (Chrysopodes) fumosus*,*Chrysopodes (Chrysopodes) spinellus* larvae have largely white to cream-colored heads with longitudinally elongate and divided, brown epicranial markings; the intermandibular and clypeal regions are unmarked. And, as in *Chrysopodes (Chrysopodes) geayi*, but not *Chrysopodes (Chrysopodes) fumosus*, the posterior ends of the frontal markings curve and connect with each other mesally. The first instar of *Chrysopodes (Chrysopodes) spinellus* differs from those of *Chrysopodes (Chrysopodes) geayi* and *Chrysopodes (Chrysopodes) fumosus* in that it usually has six thorny cranial setae (S1, S3, S4, S5, S6, S11), and the LS are amber to light brown (not dark brown or black). The *Chrysopodes (Chrysopodes) spinellus* Semaphoront B differs from both *Chrysopodes (Chrysopodes) geayi* and *Chrysopodes (Chrysopodes) fumosus* in having a thorny cranial seta S1, secondary cranial setae between S1 and S4, but no secondary sclerites on the pronotal midline.

#### First instar.

**Figure 23.**
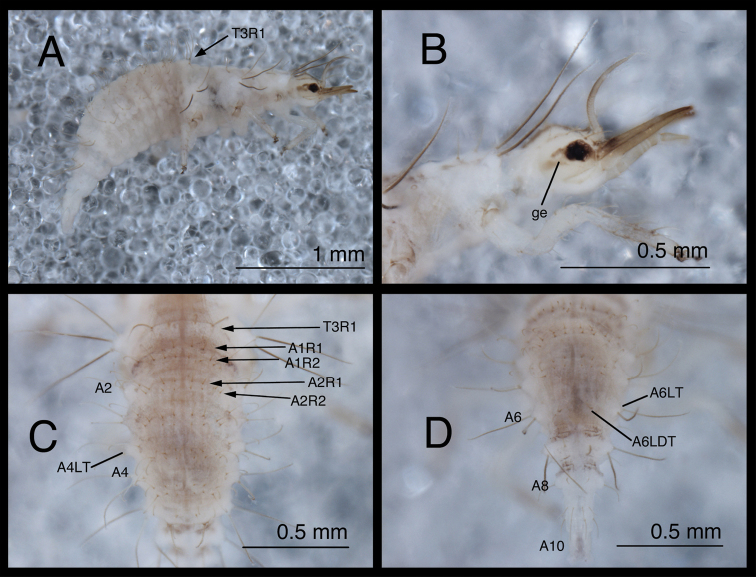
*Chrysopodes (Chrysopodes) spinellus*, first instar **A** Habitus, lateral **B** Head, lateral **C** Abdominal segments A1 to A5, dorsal **D** Abdominal segments A6 to A10, dorsal. *Abbreviations*: **A2, A4, A6, A8, A10** abdominal segments **A1R1, A1R2** anterior and posterior rows of submedian setae (SMS) on first abdominal segment **A2R1, A2R2** anterior and posterior rows of SMS on second abdominal segment **A4LT** lateral tubercle on fourth abdominal segment **A6LDT, A6LT** laterodorsal tubercle, lateral tubercle on sixth abdominal segment **ge** genal marking **T3R1** row of long, sturdy, thorny setae on raised posterior fold of metathorax.

(Semaphoront A). *Body* (Fig. 23A) 2.7–2.8 mm long; surface predominantly white to cream-colored, with some, small, light brown marks.

*Head* (Figs 2E, 23B) 0.39–0.41 mm wide; mandibles 0.36–0.37 mm long (ratio, mandible length : head width = 0.90–1.01 : 1). Cranium white, with elongate, narrow, light brown markings. Epicranial marking with lateral and mesal arms unconnected, narrow, longitudinally elongate, light brown; mesal arm contiguous with postfrontal marking, extending from base of cranium to anteromesal margin of antennal socket; lateral arm lighter brown, more diffuse than mesal arm, extending from posterolateral margin of cranial suture approximately to base of eye. Postfrontal marking indistinguishable, fused with epicranial marking (mesal arm). Frontal marking narrow, extending from midregion of cranium anteriorly to level of antennal socket, then curving laterally toward lighter brown mark at mesal margin of mandibular base; mesal ends of paired frontal marking bending mesally at tips. Intermandibular, clypeal area white. Cranial setae amber to light brown; S1, S3, S4, S5, S6, S11 thorny, robust; S1, S4, S11 long, others shorter; S2, S5 closely spaced; Vx setae small.

Gena, ventral margin of head capsule mostly white, with light brown genal mark posterior to eye, with clear spot near anterior margin. Labial palpus mostly white, middle segment tinged with light brown laterally, distal segment mostly tinged with brown. Mandibles amber to light brown, with dark brown basolateral spot. Antenna with scape light brown, pedicel white basally, brown distally; flagellum light brown.

*Thorax* (Figs 2E, 23A) mostly white, with sclerotized structures light brown to brown; episternum brown. Legs white, with base of coxa brown, distal one-fourth of tibia, basal one-half of tarsus tinged with brown; tarsal claws, empodia, brown. LS brown; other setae amber to brown.

T1: Row of three very small setae (R1) at anteromesal base of LTs. Sc1 with scattered brown areas, especially laterally; S2Sc1 small, immediately above S1Sc1. S1, S3 intermediate-length. S2, S3 thorny. T2: Spiracle with lip of atrium flush with level of integumental surface. Sc1, Sc2 transparent; Sc3 light brown; S2Sc3 medium-length to long, slender, S2 shorter. T3: S1Sc1, S2Sc1 usually present, S1Sc2 very small; S2Sc2 absent. Raised posterior fold with row of four robust, thorny, pointed setae on chalazae with ovate, light brown marks anteriorly.

*Abdomen* (Figs 23C–D) white to cream-colored, with patch of brown on dorsolateral margin of A1; anterior regions of A7, A8 marked with diffuse, scattered brown patches. LTs, LDTs white; chalazae of most dorsal setae brown.

A1-A5: Dorsum with 12 SMS in two rows, with four mesal pairs equally spaced, two lateral-most pairs juxtaposed; spiracle with SSp elongate, robust, mesal to spiracle; chalazae of all SMS and SSp not large. A6: Anterior row with two SMS, pair of small, straight setae lateral to SMS; spiracle at anterior base of LT, without apparent SSp. A7: Anterior region without setae or microsetae. Spiracle without apparent SSp. A8: Venter with two pairs of medium-length setae posteriorly.

#### Second and third instars.

**Figure 24. F24:**
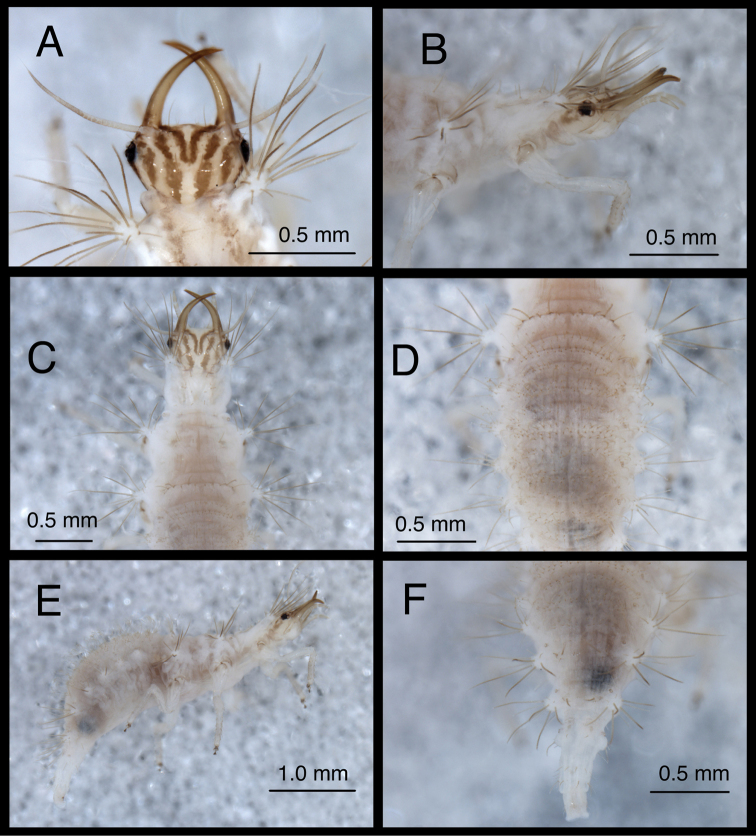
*Chrysopodes (Chrysopodes) spinellus*, second instar **A** Head, dorsal **B** Head, lateral **C** Head and thorax, dorsal **D** Abdominal segments A1 to A5, dorsal **E** Habitus, lateral **F** Abdominal segments A6 to A10, dorsal.

**Figure 25. F25:**
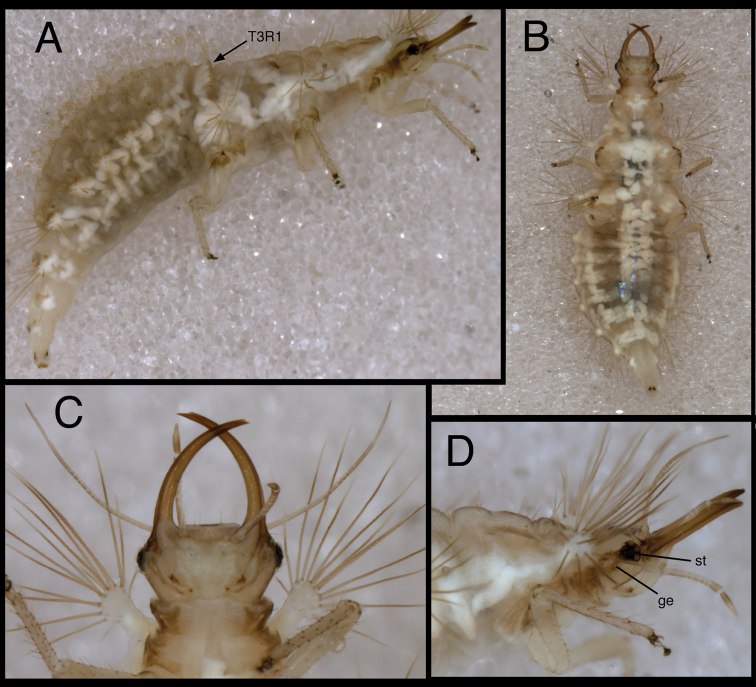
*Chrysopodes (Chrysopodes) spinellus*, third instar **A** Habitus, lateral **B** Habitus, ventral **C** Head, ventral **D** Head, lateral. *Abbreviations*: **ge** genal marking **st** stemmata **T3R1** row of long, sturdy, thorny setae on raised posterior fold of metathorax.

(Semaphoront B). *Body* (Figs 24E, 25A–B) 4.1–4.2 mm (L2), 5.8–7.0 mm (L3); surface white to tan, with light brown integumental spinules throughout; primary pronotal, mesonotal sclerites light brown; base of lateral tubercles without marks; lateral section of thorax, abdomen light brown to brown, with lateral tubercles and area below white; sclerites anterior to coxae brown.

*Head* (Figs 3E, 4E, 24A–B, 25C–D) cream-colored, with brown to dark brown markings. Epicranial marking brown, consisting of two elongate arms, separate from each other, both in contact with posterior margin of head; lateral arm extending from distolateral margin of cranium to lower level of eye, becoming narrow distally, extending to upper level of eye; mesal arm extending from base of head, contacting postfrontal marking near base of frontal marking. Postfrontal marking dark brown, robust throughout, extending to inner margin of antennal base. Frontal marking dark brown, with each arm narrow, separate (except at basal tip), extending from midsection of head, beyond tentorial pits to inner base of mandibles; base of each arm tapering, turning mesally, contacting tip of other arm. Intermandibular marking present as light brown connection between distal ends of frontal marking. Clypeolabral region beyond intermandibular marking cream-colored. Gena cream-colored, with large, brown marking from base of eye to posterior margin of cranium, with small, closed, cream-colored mesal spot distally. Mandible, maxilla amber basally, mesally, brown laterally, distally. Labial palpus: basal segment cream-colored with very slight tinge of brown; mesal segment ringed with brown laterally, cream-colored mesally, with terminal subsegment brown; terminal segment brown basally, cream-colored distally. Antenna: scape light brown, basal ~one third of pedicel cream colored, distal two-thirds of pedicel darker brown, flagellum cream-colored with slight tinge of brown. Venter cream-colored, with large, white central area; margin of cranium with light brown longitudinal marks; cardo marked with dark brown; mentum with very light brown spot basally.

Cephalic seta S1 moderately long, thorny, S2-S12 smooth, only S11 long; Vx setae moderately long; three to four pairs of small secondary setae between S1 and S4.

Head width across eyes, 0.5–0.6 mm (L2), 0.84–0.86 mm (L3); mandible length, 0.54–0.57 mm (L2), 0.86–0.90 mm (L3); ratio mandible length to head width = 0.91–0.99 : 1 (L2), 1.00–1.05 : 1 (L3). Tip of mandible with six teeth mesally.

Cervix cream-colored, tinged with light brown; sides with pair of broad brown patches; venter brown laterally, becoming cream-colored mesally; with three pairs of small setae ventrally.

**Figure 26.**
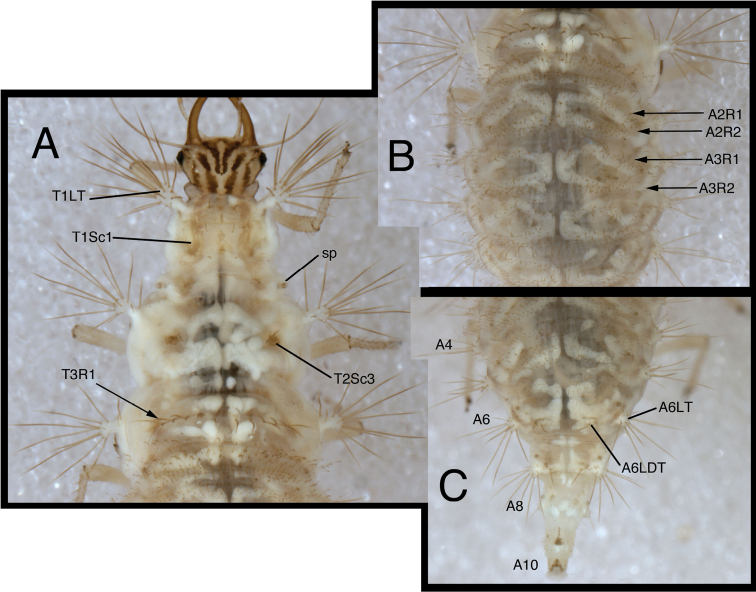
*Chrysopodes (Chrysopodes) spinellus*, third instar **A** Thorax, dorsal **B** Abdominal segments A1 to A5, dorsal **C** Abdominal segments A6 to A10, dorsal. *Abbreviations*: **A4, A6, A8, A10** abdominal segments **A2R1** double row of submedian setae (SMS) on anterior fold of second abdominal segment **A2R2** double/triple row of SMS on posterior fold of second abdominal segment **A3R1** double row of SMS on anterior fold of third abdominal segment **A3R2** double/triple row of SMS on posterior fold of third abdominal segment **A6LDT**, **A6LT** laterodorsal tubercle, lateral tubercle on sixth abdominal segment **sp** spiracle (on anterior subsegment of mesothorax) **T1LT** prothoracic lateral tubercle **T1Sc1** first primary prothoracic sclerite **T2Sc3** third primary mesothoracic sclerite **T3R1** row of long, sturdy, thorny setae on raised posterior fold of metathorax.

*Thorax* (Figs 3E, 4E, 24B-C, 24E, 25A–B, 26A) light brownish dorsally, tinged by covering of light brown spinules; sclerites, chalazae light brown; LTs white, with LS white to light amber; small tubercles beneath primary setae cream-colored to white. Venter cream-colored, with white mesal stripe, largely without marks. Legs: coxa white, with dark brown on dorsal surface; trochanter white to cream-colored, femur white, with slight tinge of brown distally; tibia white to tinged with very light brown, with light brown setae; tarsus white, tinged with very light brown; empodium, base brown; claws amber.

T1: LT with 16–17 (L2), 17–19 (L3) LS; five to six short, smooth setae anterobasally. Sc1 large, extending up mesal base of LT, light brown mesally, transparent laterally. Sc2 triangular, light brown; without secondary sclerites. S2, S3 thorny. T2: Sc1 light brown; spiracle on small protuberance. Posterior subsegment with Sc2 transparent to very light brown; Sc3 pronounced, brown. LT with 12–13 (L2), 17–19 (L3) LS. T3: LT with 11–13 (L2), 16–18 (L3) LS. Posterior fold with ten to twelve robust, thorny setae.

*Abdomen* (Figs 24D-F, 25A-B, 26B-C) dorsum cream-colored to tan, with patches of white fat body visible beneath integument throughout; chalazae of dorsal setae amber to light brown; LTs white, LS cream-colored to amber. A6 with pair of brown marks anterodorsal to LTs; A6, A7 with brown marks anterior to LDTs. A8 with pair of small, light brown marks mesal to spiracles; A9 with dark brown mark mesal to spiracles. A10 with dark brown, inverted U-shaped mark distally; light brownish laterally. Sides of A2-A5 with large, diffuse, very light brown patch below each LT; venter mostly light brown laterally, white mesally; A6-A10 mostly white ventrally; venter of A10 with pair of small, dark brown marks.

A1: Dorsum with 40–56 (L2), ~116–124 (L3) SMS in two double-triple transverse bands between spiracles. A2-A5: Dorsum with 66–84 (L2), 134–174 (L3) SMS in two broad transverse bands. LTs each with 8–11 (L2), 11–21 (L3) LS: four to nine long, robust, thorny, usually pointed LS on distal surface; remaining LS less robust, smooth, hooked in patch on dorsal surface. A6: Dorsum with transverse band of 16–18 (L2), 44–58 (L3) SMS across anterior of segment; midsection with two pairs of smooth setae, mesal pair long, hooked, lateral pair short, pointed. LT with 7–8 (L2), ~14 (L3) LS of various sizes. A7: Dorsum with three pairs of very short setae anteriorly, between spiracles. LT with 6–7 (L2), 9–12 (L3) LS of various sizes. A8: Dorsum with three pairs of very small setae between spiracles; three pairs of small setae in transverse row between LTs. Venter with four transverse rows of setae, each with three to four smooth, small to medium-length, pointed setae. A9: Dorsum with one pair of very small setae anteriorly. Middle and posterior regions with two transverse rings of setae extending around segment; each ring with ~14–16 short to medium-length setae, several in each ring robust. A10: Dorsum with one pair of small setae posterior to V-shaped anterior sclerites. Several pairs of lateral setae. Venter with ~five pairs of small setae, posterior row of microsetae anterior to terminus.

#### Egg.

At oviposition, green, with white micropyle; ovoid, 0.92 to 0.97 mm long, 0.42 to 0.44 mm wide. Stalk smooth, hyaline, 8.8 to 10.1 mm long.

#### Larval specimens examined.

Several lots, each originating from a single gravid female collected in **Brazil, Rio de Janeiro**: Campos dos Goytacazes, Parque Estadual do Desengano, Babilônia, III-27-2001, XI-22-2003 (Tauber Lot 2001:007, Albuquerque Lot 2003:023); Campos dos Goytacazes, near Parque Estadual do Desengano, Fazenda Boa Vista, V-16-2002 (Tauber Lots 2002:026, 2002:029); Campos dos Goytacazes, Distrito de Morangaba, Fazenda São Julião, X-18-2005 (Tauber Lot 2005:035).

#### Biology.

The thermal influence on rates of development and reproduction in *Chrysopodes (Chrysopodes) spinellus* will be reported elsewhere (Silva et al., in preparation).

## Supplementary Material

XML Treatment for Chrysopodes (Chrysopodes) divisus

XML Treatment for Chrysopodes (Chrysopodes) fumosus

XML Treatment for Chrysopodes (Chrysopodes) geayi

XML Treatment for Chrysopodes (Chrysopodes) lineafrons

XML Treatment for Chrysopodes (Chrysopodes) spinellus

## Appendix

Characteristics of *Chrysopodes* larvae. See the figures cited here and also Figures 5–10 in Tauber (2003). Asterisks indicate new characters or significant changes from the features listed previously (Tauber 2003).

**All Instars (Semaphoront C)**

**General**

**Setation & spiracles**

(1) setaeof four types

(a) smooth, pointed, short to long [most cephalic setae, some setae on abdominal segments A6-A10, some ventral setae, all small setae] (Fig. 1)

(b) thorny, pointed to blunt or slightly spatulate, medium length to long [some cephalic setae, most setae on thoracic lateral tubercles (LS), some primary thoracic setae, some LS on A2-A6, some setae on laterodorsal tubercles of A6, A7 (LDS), some ventral setae] (Fig. 1)

(c) smooth, hooked, long (some LS on A2, A3 of L1, some LDS on A6, A7 of L3)

(d) smooth, arising from brown chalazae, with elongate, narrow stem, laterally flattened terminal hook [abdominal submedian setae (SMS) on A1-A5, anterior region of A6] (Fig. 1)

(2) setal bases usually brown

(3) spiracles simple (with cylindrical sides, atrium not enlarged), with brownish margins

**Head**

**General**

(4) mandible with four to six small terminal “teeth” (serrations)

(5) mandibular base with one seta and dorsal row of four placoid organs (Rousset 1966)

(6) stipes elongate, narrow, folded inward on longitudinal axis

(7) stemmata black

**Cranial markings**

(8) epicranial (mesal and lateral), postfrontal, frontal markings present

(9) genal marking present, forked anteriorly or, more usually, with clear spot near anterior margin

**Cephalic setae**

(10) labrum with three pairs of setae (mesal pair long, lateral pairs shorter)

(11) all primary setae, including S12 present, S1, S11 long

(12) short, stout seta laterally on tip of pedicel

(13) long terminal seta on tip of flagellum

**Thorax**

** General**

(14) lateral tubercles (LTs) about two times longer than broad

(15) primary setae straight, with pointed tips

(16) secondary setae absent

**Prothorax**

(17) paired lateral sclerites (Sc1) large, rhomboid, marked with brown, with associated seta S1Sc1 relatively long, thorny, stemming from brown chalaza

(18) primary seta S1 thorny, stemming from brown chalaza, S5 absent

(19) venter with one pair of very small setae mesal to coxae (minute on L1)

**Mesothorax**

(20) *segment divided into two distinct subsegments: anterior subsegment with single fold (= “lobe” of Snodgrass 1935), posterior subsegment consisting of two transverse folds, separated by subtle transverse groove (folds on posterior subsegment difficult to discern on L1)

(21) anterior subsegment bearing pair of small sclerites (Sc1) anteriorly, pair of spiracles (Sp) laterally

(22) *posterior subsegment: anterior fold bearing large tubercles (LTs) laterally, pair of small sclerites (Sc2) anteriorly; posterior fold bearing large, usually brown sclerite (Sc3) on anterolateral margin (posteromesal to LTs); Sc3 with one small seta (S1Sc3) and one longer, thorny seta (S2Sc3)

(23) spiracles located on rounded, lateral protuberances, without associated setae (SSp)

(24) venter with one pair of very small setae on anteromesal margin

**Metathorax**

(25) *segment with three transverse folds; anterior two folds separated by subtle, transverse groove; dorsal surface of both folds level with mesothorax; dorsum of posterior fold prominently raised above anterior two folds, separated from anterior folds by subtle groove

(26) S1, S2 absent

(27) posterior fold with transverse row (R1) of long, robust, thorny setae arising from large chalazae, each with brown, teardrop-shaped mark on anterior surface

(28) *each transverse fold with pair of small, transparent sclerites (Sc1, Sc2, Sc3); Sc1, Sc2 (when visible) located on anterior margin of fold; Sc3 (when visible) nestled in anterolateral corner of groove above R1; Sc2, Sc3 sometimes difficult to discern (L1, L2)

(29) venter with pair of very small setae on anteromesal margin

**Abdomen**

**General**

(30) overall shape gibbous

(31) depth of A2-A5 greater than depth of thorax, width not greater than thorax

(32) A6-10 becoming narrow and tubular distally; segments not withdrawn

(33) *terminus with pair of eversible, membranous pouches (small on L1)

**Folding and submedian setae (SMS)**

(34) *A1-A5 each with two, ill-defined, transverse folds separated by small grooves; each fold bearing transverse row of SMS

(35) *A6-A10 without folding; A6 with row of SMS anteriorly, without SMS posteriorly; A7-A10 without SMS

**Tubercles and associated setae**

(36) A1 without pleural or ventral region; without lateral tubercles (LTs) or laterodorsal tubercles (LDTs)

(37) A2-A7 with LTs robust

(38) A8 with LTs round, fleshy

(39) A2-A5, A8-A10 without LDTs

(40) A6, A7 each with pair of LDTs

**Spiracles**

(41) A1-A8: spiracles (Sp) sessile

(42) A1, A6, A7: Spiracular setae (SSp) small, smooth, pointed (when present); *A2-A5 with SSp indistinguishable from SMS; A8 without SSp

**Primary setae**

(43) dorsal setae S1, S2 absent from all segments

(44) *venter with following setae: A2-A7 each with pair of sublateral setae and pair of submedian setae. Sublateral setae short, smooth, extending from small chalazae on segments A2, A3, becoming larger, smooth or thorny, stemming from larger chalazae or small tubercles on segments A4, A5, stemming from small tubercles with at least one other small seta on segments A6, A7. Submedian setae long, smooth, slender on A2, A3, short on A4-A7. Note: Occasionally, one or more of the above setae may be absent, or additional ones may be present.

**First instar (Semaphoront A)**

**Head**

**Eyes**

(1) *stemmata black, with integument between all stemmata brown to black

(2) *exterior margin of stemmata with thin, brown border

**Appendages**

(3) antenna: scape without setae

(4) mandible with four terminal “teeth” (serrations), without basal setae

(5) labial palpus: distal margin of basal segment with two setae: one lateral, one mesoventral; middle segment with two distal setae; distal segment without setae

**Venter**

(6) palpiger with two pairs of distal setae

(7) mentum with two pairs of anterior setae, one pair of posterior setae

**Thorax**

** Prothorax**

(8) lateral tubercle (LT) with two LS; one microseta between LS

(9) paired lateral sclerites (Sc1) extending anteriorly to base of LT

(10) posteromesal sclerite (Sc2) not discernible

(11) primary setae S1, S3 intermediate-length to long, thorny; S2 short, adjacent to S1Sc1, S4 absent

**Mesothorax**

(12) LT with three LS; no microseta

(13) *Sc1 with two small associated setae (S1Sc1, S2Sc1), one on each side; S3Sc1 absent. Sc2 with two small associated setae (S1Sc2, S2Sc2) laterally; S3Sc2 absent

(14) primary setae: S1 short to minute or absent, S2 intermediate-length to long, thorny

**Metathorax**

(15) *posterior fold with four to seven (perhaps eight) R1 setae

(16) *Sc1 with one to two small associated setae (S1Sc1, S2Sc1) (sometimes absent)

(17) Sc2 difficult to discern, sometimes with small associated seta (S1Sc2)

(18) Sc3 with one small associated seta (S1Sc3), sometimes absent

(19) primary setae S1, S2 not discernible

**Abdomen**

** Folding and setae**

(20) *A1-A5 each with two primary rows of SMS, sometimes lateral two SMS juxtaposed

(21) A6 with anterior area bearing single transverse row of SMS; posterior area without SMS

**Tubercles and associated setae**

(22) A2-A7: LT robust basally, tapering, sclerotized distally, bearing two long, robust setae (LS)

(23) *A2-A3: LT with one thorny, pointed LS, one smooth, hooked LS

(24) A4-A7: LT with two thorny, pointed to blunt LS

(25) A8: LT with one medium-length, thorny, pointed LS, one short, smooth LS

(26) A6, A7: LDT bearing one long, robust, thorny LDS, one short, thorny LDS (A6) or two small LDS (A7)

(27) *A6, A7: with pair of small setae between LDTs

**Spiracular setae**

(28) *A1: spiracle with or without SSp

(29) *S2-A5 with single pair of smooth, hooked SSp

(30) *A6, A7 with small, smooth, pointed SSp present or absent

(31) A8 without SSp

** Setation of terminal segments**

(32) *A9: anterior section with two pairs of robust, thorny, pointed to blunt setae dorsally, one pair laterally

(33) A9: posterior section with one pair of robust, thorny setae dorsally, one pair laterally, three pairs ventrally

(34) *A10: with scattered small setae, microsetae

**Second and third instars (Semaphoront B)**

**General**

**Integument**

(1) dorsal and ventral surface with rounded, brownish spinules

(2) spinules generally pointed, without microsetae

**Head**

**Eyes**

(3) *integument surrounding three anterior and central stemmata brown

(4) *integument around outer margin of posterior two stemmata unmarked

**Appendages (antenna & labial palpus)**

(5) scape with robust seta on distolateral margin

(6) tip of pedicel with relatively long, stout lateral seta

(7) terminal flagellar seta long

(8) basal segment of labial palpus with one small, dorsal seta, one medium-length, lateral seta, one long, ventral seta

(9) middle segment of labial palpus (L3) with ~five annulations, eight setae (three near apex of segment)

(10) middle segment of labial palpus (L2) with ~four annulations, six setae (three near apex of segment)

(11) distal segment of labial palpus without setae, with small rings throughout; ventral surface with two small, longitudinal sensory organs

**Venter**

(12) palpiger with two intermediate-length setae

(13) mentum (L3) with two pairs of long setae anteriorly below palpiger, one pair of long setae laterally above stipes, one pair of medium-length setae posteriorly

(14) mentum (L2) with two pairs of anterior setae, one pair of posterior setae

**Thorax**

**General**

(15) LTs well sclerotized, papiliform, with globose terminus

**Prothorax**

(16) paired lateral sclerites (Sc1) extending well onto the base of LT

(17) posteromesal sclerite (Sc2) present, marked with brown, triangular, smaller than Sc1

(18) *secondary sclerites present or absent

(19) S2Sc1 present, short

(20) primary setae S1 thorny, S2, S3 smooth or thorny, S4 short, smooth

(21) row of three small setae (R1) usually on anterolateral margin of notum (sometimes obscured beneath cervical fold)

**Mesothorax**

(22) Sc1 with two (L2) to three (L3) small associated setae – one mesal (S1Sc1), two lateral (S2Sc1, S3Sc1-L3 only)

(23) Sc2 with two very small associated setae, both lateral (S1Sc2, S2Sc2)

(24) *Sc3 with two associated setae – S1Sc3 short, smooth, S2Sc3 intermediate-length to long, thorny

(25) *primary setae S1, S2 smooth, short to medium-length, anterior to Sc3; S3 robust, thorny, intermediate length to long, usually located in transverse row with S2Sc3; S4 small when present, often absent

**Metathorax**

(26) *Sc1, Sc2, Sc3 each with pair of sclerites bearing one to two small, associated setae (S1Sc1, S2Sc1, S1Sc2, S2Sc2, S1Sc3 sometimes absent)

(27) posterior fold with ten to 16 robust, thorny R1 setae

**Abdomen**

(28) *A1-A5: dorsum with transverse folds spinule-covered, separated by shallow groove without spinules

(29) each fold bearing double or triple transverse band of submedian setae (SMS)

(30) A2-A7: LTs hemispherical to slightly flattened apically, with brown marks anteriorly and posteriorly (sometimes light, sometimes absent from L2)

(31) *A2-A5: LTs each with one to eight long, thorny, robust, blunt to spatulate LS apically (size, thorniness, robustness of these LS increasing posteriorly), with subapical dorsal setae long, smooth, hooked, similar to SMS

(32) A6, A7: all LS long, thorny, robust, blunt to spatulate, restricted to apical region of LT

(33) A8: LT bearing one medium-length, thorny, pointed seta (LS), one or more short, smooth LS

(34) A6, A7 with LDTs, each bearing one to two long, robust, pointed or blunt (not hooked), thorny setae (LDS) stemming from large, brown chalazae, sometimes a long, smooth, hooked LDS, and three to four smaller, smooth or thorny LDS

(35) A6, A7 with pair of short, pointed setae between LDTs

(36) A1-A5 with patches of setae near or around each spiracle [These setae are indistinguishable in size and shape from SMS, and the patches often merge with the anterior rows of SMS. Thus, here, they are included with the SMS.]

(37) A6, A7, A8 with single pair of small, pointed SSp
